# Perceived barriers and facilitators to mental health help-seeking in young people: a systematic review

**DOI:** 10.1186/1471-244X-10-113

**Published:** 2010-12-30

**Authors:** Amelia Gulliver, Kathleen M Griffiths, Helen Christensen

**Affiliations:** 1 Centre for Mental Health Research, The Australian National University, Canberra, Australia

## Abstract

**Background:**

Adolescents and young adults frequently experience mental disorders, yet tend not to seek help. This systematic review aims to summarise reported barriers and facilitators of help-seeking in young people using both qualitative research from surveys, focus groups, and interviews and quantitative data from published surveys. It extends previous reviews through its systematic research methodology and by the inclusion of published studies describing what young people themselves perceive are the barriers and facilitators to help-seeking for common mental health problems.

**Methods:**

Twenty two published studies of perceived barriers or facilitators in adolescents or young adults were identified through searches of PubMed, PsycInfo, and the Cochrane database. A thematic analysis was undertaken on the results reported in the qualitative literature and quantitative literature.

**Results:**

Fifteen qualitative and seven quantitative studies were identified. Young people perceived stigma and embarrassment, problems recognising symptoms (poor mental health literacy), and a preference for self-reliance as the most important barriers to help-seeking. Facilitators were comparatively under-researched. However, there was evidence that young people perceived positive past experiences, and social support and encouragement from others as aids to the help-seeking process.

**Conclusions:**

Strategies for improving help-seeking by adolescents and young adults should focus on improving mental health literacy, reducing stigma, and taking into account the desire of young people for self-reliance.

## Background

### The burden and prevalence of mental disorders

Depression and anxiety are highly prevalent mental disorders with estimates indicating they affect up to almost one fifth of the population in high income countries worldwide [1-3]. Prevalence of mental disorders is greatest among younger people aged 16-24 years [4] than at any other stage of the lifespan. They are also common in childhood and adolescence with 14% of those aged between 4 and 17 years affected [5]. This high susceptibility in adolescents and young adults to developing a mental disorder is coupled with a strong reluctance to seek professional help [6].

### Reluctance to seek help

Studies have found that approximately 18 to 34% of young people with high levels of depression or anxiety symptoms seek professional help. For example, a school-based study of 12 to 17 year old German adolescents reported that only 18.2% of those with diagnosable anxiety disorders, and 23% of those with depressive disorders had ever used mental health services [7]. Similarly, a large study of over 11,000 Norwegian adolescents in school aged 15 to 16 years found that only 34% of those with high levels of depression and anxiety symptoms had sought professional help in the previous year [8]. According to an Australian national mental health survey of young people only 25% of children aged 4 to 17 years with a diagnosable mental disorder had used any health services in the 6 months prior to the survey [5]. This reluctance to seek help is not limited to children and adolescents. Adults of all ages often do not seek help for a mental illness [9], with only 35% of those surveyed with a common mental disorder seeking help during the previous year [4].

### Proposed reasons for not seeking help

Many reasons have been proposed to explain why adults in the general population do not seek professional help for common mental disorders. These include negative attitudes towards seeking help generally [10], as well as concerns about cost, transportation or inconvenience, confidentiality, other people finding out, feeling like they can handle the problem on their own, and the belief that the treatment will not help [11]. Similar concerns have been found in a rural population, with the addition of worry that that the care will be unavailable when needed, about being treated unkindly, and not knowing where to go [12]. Conversely, facilitators have been proposed to include prior treatment, higher education, and greater mental disorder episode length [13], and the influence of intimate partners and general practitioners [14].

Likewise, research has sought to explain the reluctance of young people and adolescents to seek professional help when it is necessary. Friends and family are often the preferred sources of help over health professionals [6,15]. In two reviews of help-seeking studies, Rickwood and her collaborators concluded that a high reliance on self to solve problems, a lack of emotional competence, and negative attitudes about seeking professional help were barriers to help-seeking [6,16]. Conversely, the authors identified a number of possible facilitators of help-seeking. These included emotional competence, knowledge, positive attitudes towards seeking professional help, social encouragement, and the availability of established and trusted relationships with professionals such as general practitioners [6]. These reviews were based around a model of help-seeking [16] in which seeking professional help is conceptualised as a multi-step process beginning with the individual's development of an awareness of the problem, followed by the expression of the problem and a need for help to others, the identification of appropriate of sources of help for the individual to access, and finally, the willingness of the individual to actually seek out and disclose to potential sources of help. In another review, Barker and colleagues [17] differentiated between structural and personal determinants of help-seeking. They maintained that individual factors, such as personal beliefs, internalised gender norms, coping skills, self-efficacy, and perceived stigma interact with structural factors including the national health system, accessibility and affordability of services, and social support. However, none of these reviews were systematic syntheses of the available quantitative and qualitative literature. Moreover, they focused primarily on quantitative cross-sectional correlational studies (e.g., primarily survey studies which measured the association between a measured barrier such as low emotional competence and the young person's intentions to seek help [18]) and largely overlooked the qualitative research. The qualitative research in particular may provide additional and different information about the reasons that young people do not seek help to structured survey questions. Moreover, currently no review has systematically identified and synthesised the literature which asks young people themselves what they perceive are the barriers and facilitators to help-seeking. This systematic review seeks to address this gap.

### Aims and scope of this study

This study is a systematic review of both the qualitative and the quantitative literature on the perceived barriers and facilitators to help-seeking for mental health problems in adolescents and young adults. In this paper 'adolescents' refers to those aged between 12 and 17 years and 'young adults' to those aged 18 to 25 years [19]. It focuses on help-seeking for the common mental health problems of depression, anxiety and general emotional distress.

## Methods

### Databases & Search methodology

Three databases (PubMed, PsycINFO, and Cochrane) were searched in September and October 2009 using the search terms presented in additional file 1: Search terms. These terms aimed to represent the primary concepts of 'help-seeking', 'mental health', and 'barriers' or 'facilitators'. Keywords were generated for each of these concepts by examining the terminology used in review papers in the help-seeking literature and a thesaurus to locate synonyms. In addition, the keywords were combined with standard MeSH terms from the PubMed and Cochrane databases and Subject Headings for the PsycINFO database.

### Study Selection

**Figure 1:**
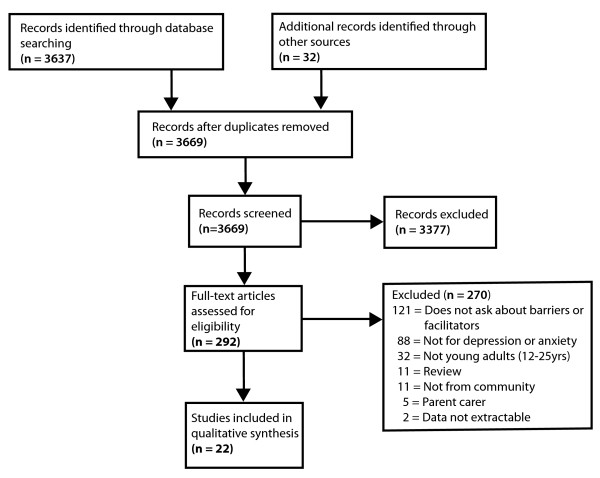
**Study selection flow diagram**.

Figure 1 presents the flow chart for the selection of the included studies. The initial database search returned 3637 published English-language abstracts after removing duplicates. One of the researchers (AG) then screened the abstracts and excluded studies that did not address barriers or facilitators to help-seeking for a mental health problem. This resulted in 260 potentially relevant studies. An additional 32 studies were located through hand-searching the reference lists of reviews and key papers found through the systematic search and which were considered likely to satisfy the inclusion criteria.

The second stage of the study selection process involved examining each of the 292 articles and excluding those which met the following exclusion criteria.

1. Participants were not explicitly asked what they considered were barriers or facilitators to help-seeking for mental health problems.

2. Study participants were neither adolescents nor young adults (aged 12-25 years). This criterion was considered met if more than 25% of the participants fell outside the 12 to 25 years age range, the sample mean age was 26 years or more, the sample was specifically described as "adults" with the age of the participants no further described.

3. Study was a review.

4. Study participants were not members of the general community, or university, or school students (e.g., studies of groups with restricted access to a range of help-seeking opportunities such as prisoners and members of the military).

5. Study was focused on help-seeking on behalf of another person (e.g., carer seeking help for a consumer, or parent seeking help for a child).

6. Study contained no extractable data on barriers or facilitators.

7. Study addressed a mental health condition other than depression, or anxiety, or general "mental distress" (e.g., psychosis, obsessive compulsive disorder). Studies of pre- or post-natal depression were also excluded.

270 studies met one or more of these criteria and were excluded from further consideration. No studies were excluded on the basis of research quality. A summary of the excluded studies grouped by the primary reason for the exclusion is provided in additional file 2: List of studies excluded from the review by exclusion category. This process resulted in a total of 22 relevant studies [20-41] for inclusion into the review.

### Coding of Studies

Each of the 22 included studies was coded using a pre-formulated rating sheet according to the following characteristics: Author name, year published, country (location of study), age of participants (age range or mean), population description, whether the study participants were selected regardless of mental health status or risk profile (universal) or not, sample size (for target variable), gender (male, female, both), setting (e.g., high school, community, university), research type (qualitative, quantitative), specified barriers to help-seeking (description of barrier themes or items as listed in the study), and specified facilitators of help-seeking (description of facilitator themes or items as listed by the study).

### Analysis strategy

Standard methods for thematic analysis [42] were conducted on participant reported barriers and facilitators in the qualitative studies. Barriers and facilitators reported in the quantitative studies were tabulated and top rated themes extracted.

## Results

### 1. Study characteristics

The characteristics of each of the qualitative and quantitative studies of the perceived barriers and facilitators of help-seeking in young people are detailed in additional file 3: Qualitative studies included in the review; and additional file 4: Quantitative studies included in the review. The following section provides an overview of these characteristics including the year and location of the study, the methodologies employed, and the characteristics of the study participants.

#### Year and location of studies

The studies were published between 1990 and 2008 with most conducted in the Australia (n = 10), or the United States (n = 9). A further two studies were undertaken in the United Kingdom, and one in China.

#### Methodologies employed

The majority of studies were conducted using qualitative methods (n = 15), the remainder being quantitative studies. All seven quantitative studies used a survey method to collect data. However, the methodology employed in the 15 qualitative studies varied: seven involved interviews, four used focus groups, three used a survey method to collect data, and one employed both focus groups and interviews.

#### Sample and participant characteristics

##### Sample size

The number of participants in the studies varied markedly from 3 to 3746. The majority of the qualitative studies (n = 12 of 15) employed between 3 and 52 participants, and a further three involved between 326 and 3746 participants. Sample sizes for the seven quantitative studies ranged from 71 to 294.

##### Participant age

Data reported on the age of participants varied. Many studies provided an age range in years (11-17 to 18-31+) or the mean age of participants (15.4-21.2). However, some studies reported the grade of the participants only, and these ranged from grades 7 to 12.

##### Gender

Most studies included both males and females (n = 19). However, two focused exclusively on males and one on females only.

##### Settings and target groups

Half of the included studies (n = 11) were conducted in a high school setting. Of these, one examined Caucasian male students, and one, rural students. Five studies took place in universities. Of these, two out of five examined medical students specifically, and one recruited rural psychology undergraduates. Five studies were undertaken in a community setting, of which one examined at-risk African American male adolescents, and another investigated rural adolescents. Finally, one study was undertaken in both a high school and a community setting [41]. Overall, three of the studies involved a rural population.

##### Mental health status of participants

Most studies (n = 14) were conducted with samples not selected on the basis of participant mental health status. However, three studies focused on participants with self reported depression, another two focused on self-reported depression and/or anxiety, and the final three included participants with general 'mental distress', 'a mental health issue', or a 'health related problem', the latter from which only data for participants experiencing self-reported depression were included in the present review [26].

### 2. Perceived barriers and facilitators

#### Analysis of qualitative studies

Fifteen of the qualitative studies identified participant perceived barriers and facilitators to help-seeking for mental health problems. Two [30,33] studies were excluded from this formal analysis as rather than examining help-seeking more generally, they only addressed characteristics of school-based personnel that may aid or deter help-seeking. A meta-thematic analysis of the remaining 13 papers was conducted by collating and coding data into themes developed from terminology used by the reviewed literature. Topics specified as barriers or facilitators to help-seeking in the papers were coded respectively under thirteen different barrier and seven facilitator themes. For the detailed findings of this thematic analysis see additional file 5: Thematic analysis of qualitative studies.

*(a). Barrier themes: *Table 1 summarises the key barrier themes emerging from the analysis in order of frequency of studies in which the theme was addressed. The most frequently mentioned barrier was stigma which was reported in over three-quarters of the studies. In addition, almost half of the studies cited issues related to confidentiality and trust. Over one-third of studies referred to difficulties with identifying symptoms, concern about the characteristics of the provider, and reliance on self as perceived barriers to help-seeking.

**Table 1:** Key barrier themes and number of studies (n = 13) in which theme addressed

#	Barrier theme	Number of studies
1	Public, perceived and self-stigmatising attitudes to mental illness	10

2	Confidentiality and trust	6

3	Difficulty identifying the symptoms of mental illness	5

4	Concern about the characteristics of the provider	5

5	Reliance on self, do not want help	5

6	Knowledge about mental health services	4

7	Fear or stress about the act of help-seeking or the source of help itself	4

8	Lack of accessibility, e.g., time, transport, cost	4

9	Difficulty or an unwillingness to express emotion	3

10	Do not want to burden someone else	2

11	Prefer other sources of help (e.g., family, friends)	2

12	Worry about effect on career	1

13	Others not recognising the need for help or not having the skills to cope	1

*(b). Facilitator themes: *Few of the qualitative studies addressed the perceived facilitators of mental health help-seeking. Accordingly, only a limited analysis was possible. Table 2 details the eight facilitator themes raised in the three studies included in this analysis. Positive past experiences were mentioned by all papers examining facilitators, and it was also the theme for which the greatest number of individual facilitators was reported.

**Table 2:** Key facilitator themes and number of studies (n = 3) in which theme addressed

#	Facilitator theme	Number of studies
1	Positive past experiences with help-seeking	3

2	Social support or encouragement from others	2

3	Confidentiality and trust in the provider	2

4	Positive relationships with service staff	2

5	Education and awareness	1

6	Perceiving the problem as serious	1

7	Ease of expressing emotion and openness	1

8	Positive attitudes towards seeking help	1

#### Analysis of quantitative studies

None of the seven quantitative studies addressed facilitators. Each of these studies used a survey method to elicit respondent views about relevant barriers (i.e., responses to barrier scales, endorsing barriers from a list, and rating the relative importance of barriers).

*(a). Barrier themes: *The list of potential barriers rated by participants in the quantitative studies varied across studies. The top rated barriers (i.e., those endorsed by the greatest percentage of respondents or achieving the highest mean rating) are detailed in Table 3. The most commonly endorsed included *stigma *and *discomfort *discussing mental health problems, a preference for *relying on self*, and a *failure to perceive a need for help*. Other top rated barriers from the quantitative studies were believing that no one could help [26], not liking to disclose personal matters to a stranger [37], and not feeling comfortable talking to a general practitioner whom the young person did not know [38].

**Table 3:** Top rated barriers by quantitative studies (n = 7)

Author	Top rated barriers
Sheffield (2004) [35]	*School counsellor*
	1. Prefer to handle myself (45%) (self-reliance)
	2. Don't think they can help (27%) (no one can help)
	*Doctor*
	1. Too expensive (25%) (cost)
	2. Prefer to handle myself (23%) (self-reliance)
	*Psychologist/Psychiatrist*
	1. Too expensive (50%) (cost)
	2. Don't know where to find (28%) (knowledge)

Dubow (1990) [26]	1. I felt that no person or helping service could help (55%) (no one can help)
	2. The problem was too personal to tell anyone (53%) (stigma/comfort)

West (1991) [37]	1. I do not like to tell a stranger about personal things (29.4%) (stigma/comfort)
	2. I am afraid counsellor will pass information about me to other people (18.3%) (confidentiality)

Kuhl, (1997) [32]	1. If I had a problem I would solve it by myself (3.87) (self-reliance)
	2. I think I should work out my own problems (3.79) (self-reliance)

Wilson (2008) [38]	1. I feel comfortable talking to a GP (general practitioner) who I don't know (1.65) (stigma/comfort)
	2. I'm not embarrassed to talk about my problems (1.51) (stigma/comfort)

Eisenberg (2007) [27]	1. Stress is normal in graduate school (51%) (self-reliance)
	2. Have not had any need (45%) (no perceived need)

Brimstone (2007) [24]	1. Worries about either knowing the doctor/counsellor or having to have future dealings with the counsellor/psychologist or general practitioner at university health care centre (stigma/comfort)
	2. Worries about either knowing the doctor/counsellor or having to have future dealings with the counsellor/psychologist or general practitioner at non-university health care centre (stigma/comfort)

## Discussion

The present review identified a range of perceived barriers and facilitators to help-seeking. However, it is clear from the present systematic review that there is a paucity of high quality research in the area, little emphasis on identifying facilitators, and a focus on qualitative rather than quantitative data collection. The following discussion considers the most prominent barrier and facilitator themes from the systematic review, defined as those with at least five or more barriers or facilitators in the qualitative thematic analysis, and places them in the context of previous reviews and related studies in the literature.

### Prominent barrier themes

#### Public, perceived and self-stigmatising attitudes to mental illness

In the present study stigma and embarrassment about seeking help emerged in both the qualitative and quantitative studies as the most prominent barrier to help-seeking for mental health problems. This finding is consistent with conclusions from previous reviews of help-seeking in this age group [16,17]. It is of interest that all three studies focusing on rural populations [20,23,28] mentioned a high rate of barriers related to stigma, which is consistent with a previous finding that perceived stigma may affect help-seeking more in rural than urban residing adults [43]. Another study of community-based young people [31] also reported many stigma-related barriers to help-seeking from specific sources (e.g., doctor, counsellor etc.). Most of these were concerns about what others, including the source of help itself, might think of them if they were to seek help.

#### Confidentiality and trust

A major concern for many of the study participants was confidentiality and trust with respect to the potential source of help. This concern has been identified as a barrier in previous reviews [6,16] which report that young people show greater help-seeking intentions towards trusted sources. Concern about confidentiality and trust may also relate to stigma, where a fear of a breach in confidentiality stems from the fear of stigma and embarrassment should peers and family find out that the young person had sought help.

#### Difficulty identifying the symptoms of mental illness

A lack of insight into or understanding of symptoms has been discussed previously in the context of help seeking in cross-sectional correlational studies [15] and reviews [16]. One study [21] of young people with mental distress reported that participants were aware of their distress, but continuously altered the meaning they attached to this distress, and in particular whether or not it was "normal" in order to accommodate higher levels of distress and avoid seeking help.

#### Lack of accessibility

Lack of accessibility (e.g., time, transport, cost) was a prominent barrier particularly in the studies of rural populations, a finding which is consistent with previous research on adults in rural areas [12]. In rural settings where there is a paucity of mental health professionals, young people may find it difficult to source close by and available help.

#### Self-reliance

Both the qualitative and quantitative research in the present study indicated that adolescents and young adults prefer to rely on themselves rather than to seek external help for their problems. Again, this common barrier to help-seeking has also been reported in previous reviews of cross-sectional studies [6]. In addition, previous research suggests that adolescent preferences for self-reliance during difficult times, extends to a preference for self-help as a treatment for mental health difficulties [44].

#### Concern about characteristics of provider

Some of the studies in the review found that the characteristics of the potential provider of help (e.g., psychologist, general practitioners etc.) could be deterrents to seeking help. This included features such as race, the ability of the provider to provide help, their credibility, and whether they were known to the young person. Though they were not incorporated into the thematic analysis, two studies [30,33] reported the qualities of potential providers in schools that young people perceived as barriers to help-seeking. These were active negativity ("rude and smart aleck"), breach of confidentiality ("not enough privacy in school"), dual roles ("hard to talk to somebody when you think of them as an enforcer of the school rules"), judgmental attitude or tendency to show favouritism ("some adults don't see both sides"), unhelpful responses ("they blow it out of proportion-exaggerate"), being out of touch with adolescents ("they don't know about gangs and drugs"), psychologically inaccessible ("never assure you that you can come and talk to them"), and too busy ("they have too many kids to deal with"). These two studies also emphasise that young people place importance on the characteristics of the person potentially providing the help.

#### Knowledge about mental health services

Young peoples' lack of knowledge about mental health services was also a perceived barrier to help-seeking, a finding which is consistent with prior reviews [6,16,17]. In particular, study participants did not consider a general practitioner an appropriate source of help for mental distress. This has been found previously in a qualitative research study using interviews to investigate young peoples' attitudes towards general practitioners as a source of help [45].

#### Fear or stress about the act of help-seeking or source of help itself

Many young people reported that they were fearful about the act of seeking help, or the source of help itself. Consistent with this theme, there is evidence that young people who have established relationships with health professionals are more likely to seek help in the future [16]. Thus experience with sources of help may reduce fears about the unknown, and encourage young people to seek further help.

#### Prominent facilitator theme

##### Positive past experiences

All three studies investigating facilitators reported positive past experiences [34,36,40] as a facilitator of help-seeking in their samples of high school students. Past experience with help-seeking may also act as a form of knowledge or mental health literacy, a topic deemed important in the help-seeking process [6,46].

#### Limitations

Several limitations to the present study need to be considered. First, the search strategy may not have captured all of the relevant articles. The choice of database influences the coverage of potential journal papers to be included [47]. This review employed only three databases; some relevant journals may not have been indexed by these databases. Further, the terminology utilised in the search strategy may not have been sufficiently broad to capture all published research on barriers and facilitators in young people. However, this must be balanced against the feasibility of processing the results of an over-inclusive search strategy. Hand-searching of reference lists located some further papers not captured in the database searches [48]. A final limitation of the search strategy was that for practical reasons only published literature was sourced; however, it seems unlikely that publication status would be a substantial source of bias in the current context.

Another limitation is that only one researcher coded the retrieved barriers and facilitators into themes and as such the coding of themes may be biased. Qualitative research is by its nature a subjective process. For the purposes of transparency, the current paper provides details of the data from which the themes were extracted in the qualitative analysis.

A further limitation of the study is that this review utilised counts of themes and the number of studies reporting each theme in the qualitative research, as well as the highest-rated barriers and facilitators in the quantitative research. Although such counts may reflect the relative importance of topics we acknowledge that this is not necessarily the case. For example, it may overemphasise the importance of topics which were mentioned in various different forms (e.g., self-stigma, social stigma). However, the method provides a useful starting point for generating future research and particularly for suggesting potential appropriate targets for intervention to increase help-seeking.

Finally, it is a limitation that this study addresses only those perceived barriers and facilitators to help-seeking reported by young people given that they may not be aware of all the potentially influential factors.

## Conclusions

Young people perceive a number of barriers to help-seeking for mental health problems. These include stigma and embarrassment, problems recognising symptoms (poor mental health literacy), and a preference for self-reliance. These were prominent themes in both the qualitative and quantitative literature. Less is known about those factors which young people believe facilitate help-seeking. However, there is some evidence that positive past experiences, which may increase mental health literacy, as well as social support and encouragement from others, which may reduce the stigma of help-seeking, are facilitators of help-seeking in this age group. The findings suggest a number of ways forward. First, strategies for improving mental health among young people need to address the young person's desire for self-reliance. One potential approach involves the provision of evidence-based self-help material. A second involves providing a program to increase the young person's mental health literacy, and in particular to increase their knowledge of their own symptoms. A final approach involves the provision of programs to young people that are designed to reduce the stigma associated with mental illness and mental health help-seeking. Nevertheless, barriers and facilitators may vary across the different points of the help-seeking process, and a more sophisticated investigation of these factors as they operate at each level of the help-seeking process is required to advance the field.

This systematic review conforms to the PRISMA statement [49]. A PRISMA checklist is provided in additional file 6: PRISMA 2009 Checklist.

## Competing interests

The authors declare that they have no competing interests.

## Authors' contributions

AG designed the study and the search criteria, developed coding checklists, coded the papers, undertook the analyses and wrote a draft of the manuscript. KG and HC supervised all stages of the research, and contributed to the design and analysis of the study and edited the paper. All authors read and approved the final manuscript.

## Pre-publication history

The pre-publication history for this paper can be accessed here:

http://www.biomedcentral.com/1471-244X/10/113/prepub

## Supplementary Material

Additional file 1 **Search terms**. Click here for file

Additional file 2 **List of studies excluded from the review by exclusion category**. Click here for file

Additional file 3 **Qualitative studies included in the review**. Click here for file

Additional file 4 **Quantitative studies included in the review**. Click here for file

Additional file 5 **Thematic analysis of qualitative studies**. Click here for file

Additional file 6 **PRISMA 2009 Checklist**. Click here for file
