# Supplementary material for: Perceived barriers and facilitators to mental health help-seeking in young people: a systematic review
Source: BMC Psychiatry. 2010 Dec 30;10:113. doi: 10.1186/1471-244X-10-113 (PMC3022639; doi:10.1186/1471-244X-10-113)
Supplement: Additional file 1 — Search terms. [file 1471-244X-10-113-S1.DOC]

**Search terms**

***SEARCH 1: PsycINFO (conducted 23/09/09)***

1. Barrier$ OR Hurdle OR Promot$ OR Obstruct$ OR Facilitat$ OR Support$ OR Cause$ OR Encourag$ OR Treatment Barriers (Subject Heading)

**AND**

2. Mental Health (Subject Heading) OR Mental Disorders (Subject Heading)

**AND**

3. Helpseek$ OR Seek$ help OR Seek$ treatment OR Help Seeking Behavior (Subject heading)

**Limits:** Humans and (adolescence “age 13 to 17 yrs” or young adulthood “age 18 to 29” yrs or thirties “age 30 to 39 yrs” or middle age “age 40 to 64 yrs”)

***SEARCH 2: PubMed (conducted 22/10/09)***

1. Barrier* OR Hurdle OR Promot* OR Obstruct* OR Facilitat* OR Support* OR Cause* OR Encourag* OR Health behavior [MeSH]

**AND**

2. Mental Health OR Mental disorder [MeSH]

**AND**

3. Help seek* OR Seek* help OR Seek* treatment

**Limits:** **Humans, Adolescent: 13-18 years, Adult: 19-44 years**

***SEARCH 3: Cochrane (conducted 23/10/09)***

1. Barrier* OR Hurdle OR Barricade* OR Obstruct* OR Facilitat* OR Supporter* OR Cause* OR Health behavior [MeSH]

**AND**

2. Mental Health OR Mental health [MeSH]

**AND**

3. Help seek* OR Seek* help OR Seek* treatment

**Limits:** None
