# Supplementary material for: Perceived barriers and facilitators to mental health help-seeking in young people: a systematic review
Source: BMC Psychiatry. 2010 Dec 30;10:113. doi: 10.1186/1471-244X-10-113 (PMC3022639; doi:10.1186/1471-244X-10-113)
Supplement: Additional file 2 — List of studies excluded from the review by exclusion category. [file 1471-244X-10-113-S2.DOC]

**List of studies excluded from the review by exclusion category**

Studies were excluded because they: (1) did not investigate adolescent/young adult perceived barriers and facilitators (n=121), (2) did not involve adolescents/young adults (n=32), (3) were reviews (n=11), (4) were focused on settings outside the general community (n=11; 7 prisons, 4 military), (5) were focused on a parent or carer (n=5), (6) did not contain extractable data (n=2), or (7) were focused on disorders other than depression or anxiety (n=88; drug and alcohol=44, pre- or post-natal=11, psychotic disorders or schizophrenia=11; eating disorders=6, gambling=4, obsessive compulsive disorder (n=3), comorbidity or general health care=3, insomnia=1, Dementia of the Alzheimer’s type =1, orofacial injury=1, sexual dysfunction=1, intimate partner violence=1, externalising disorder=1).

The following list details these excluded studies according to the primary reason for their exclusion.

**Criterion 1. Did not investigate barriers and facilitators (n=121)**

| 1 | Aalto-Setala, T., Marttunen, M., Tuulio-Henriksson, A., Poikolainen, K. and Lonnqvist, J. (2002). Psychiatric treatment seeking and psychosocial impairment among young adults with depression. Journal of Affective Disorders, 70 (1), 35-47. |
| --- | --- |
| 2 | Amstadter, A. B., McCauley, J. L., Ruggiero, K. J., Resnick, H. S. and Kilpatrick, D. G. (2008). Service utilization and help seeking in a national sample of female rape victims. Psychiatric Services, 59 1450-1457. |
| 3 | Angermeyer, M. C., Matschinger, H. and Riedel-Heller, S. G. (1999). Whom to ask for help in case of a mental disorder? Preferences of the lay public. Social Psychiatry and Psychiatric Epidemiology, 34 (4), 202-10. |
| 4 | Angermeyer, M. C., Matschinger, H. and Riedel-Heller, S. G. (2001). What to do about mental disorder--help-seeking recommendations of the lay public. Acta Psychiatrica Scandinavica, 103 (3), 220-5. |
| 5 | Bergeron, E., Poirier, L. R., Fournier, L., Roberge, P. and Barrette, G. (2005). Determinants of service use among young Canadians with mental disorders. Canadian Journal of Psychiatry, 50 (10), 629-36. |
| 6 | Biddle, L., Donovan, J. L., Gunnell, D. and Sharp, D. (2006). Young adults' perceptions of GPs as a help source for mental distress: a qualitative study. British Journal of General Practice, 56 (533), 924-31. |
| 7 | Biddle, L., Gunnell, D., Sharp, D., & Donovan, J. L. (2004). Factors influencing help seeking in mentally distressed young adults: a cross-sectional survey. *British Journal of General Practice, 54*(501), 248-253. |
| 8 | Blanco, C., Okuda, M., Wright, C., Hasin, D. S., Grant, B. F., Liu, S. M. and Olfson, M. (2008). Mental health of college students and their non-college-attending peers: results from the National Epidemiologic Study on Alcohol and Related Conditions. Archives of General Psychiatry, 65 (12), 1429-37. |
| 9 | Boldero, J. and Fallon, B. (1995). Adolescent help-seeking: What do they get help for and from whom? Journal of Adolescence, 18 (2), 193-209. |
| 10 | Brown, B. B. (1978). Social and psychological correlates of help-seeking behavior among urban adults. American Journal of Community Psychology, 6 (5), 425-39. |
| 11 | Bucholz, K. K. and Dinwiddie, S. H. (1989). Influence of non-depressive psychiatric symptoms on whether patients tell a doctor about depression. American Journal of Psychiatry, 146 (5), 640-4. |
| 12 | Buston, K. (2002). Adolescents with mental health problems: what do they say about health services? Journal of Adolescence, 25 (2), 231-42. |
| 13 | Carlton, P. A. and Deane, F. P. (2000). Impact of attitudes and suicidal ideation on adolescents' intentions to seek professional psychological help. Journal of Adolescence, 23 (1), 35-45. |
| 14 | Chen, S. X. and Mak, W. W. S. (2008). Seeking professional help: Etiology beliefs about mental illness across cultures. Journal of Counseling Psychology, 55 (4), 427-441. |
| 15 | Cheung, F. M. (1984). Preferences in help-seeking among Chinese students. Culture, Medicine and Psychiatry, 8 (4), 371-80. |
| 16 | Christensen, H., Leach, L. S., Barney, L., Mackinnon, A. J. and Griffiths, K. M. (2006). The effect of web based depression interventions on self reported help seeking: randomised controlled trial [ISRCTN77824516]. BMC Psychiatry, 6 13. |
| 17 | Ciarrochi, J., Wilson, C. J., Deane, F. P. and Rickwood, D. (2003). Do difficulties with emotions inhibit help-seeking in adolescence? The role of age and emotional competence in predicting help-seeking intentions. Counselling Psychology Quarterly, 16 (2), 103-120. |
| 18 | Ciarrochi, J. and Deane, F. (2001). Emotional competence and willingness to seek help from professional and nonprofessional sources. British Journal of Guidance and Counselling, 29 (2), 233-246. |
| 19 | Ciarrochi, J., Deane, F., Wilson, C. and Rickwood, D. (2002). Adolescents who need help the most are the least likely to seek it: the relationship between low emotional competence and low intention to seek help. British Journal of Guidance and Counselling, 30 (2), 173-188. |
| 20 | Claassen, C. A., Hughes, C. W., Gilfillan, S., McIntire, D., Roose, A. and Basco, M. (2000). The nature of help-seeking during psychiatric emergency service visits by a patient and an accompanying adult. Psychiatric Services, 51 (7), 924-7. |
| 21 | Cotton, S. M., Wright, A., Harris, M. G., Jorm, A. F. and McGorry, P. D. (2006). Influence of gender on mental health literacy in young Australians. Australian and New Zealand Journal of Psychiatry, 40 (9), 790-6. |
| 22 | Deane, F. and Todd, D. (1996). Attitudes and intentions to seek professional psychological help for personal problems or suicidal thinking Journal of College Student Psychotherapy, 10 (4), 45-59. |
| 23 | Deane, F. and Chamberlain, K. (1994). Treatment fearfulness and distress as predictors of professional psychological help-seeking. British Journal of Guidance and Counselling, 22 (2), 207-217. |
| 24 | Deane, F., Wilson, C. and Ciarrochi, J. (2001). Suicidal ideation and help-negation: not just hopelessness or prior help. Journal of Clinical Psychology, 57 (7), 901-914. |
| 25 | Dejman, M., Ekblad, S., Forouzan, A. S., Baradaran-Eftekhari, M. and Malekafzali, H. (2008). Explanatory model of help-seeking and coping mechanisms among depressed women in three ethnic groups of Fars, Kurdish, and Turkish in Iran. Archives of Iranian Medicine, 11 (4), 397-406. |
| 26 | Dew, M. A., Bromet, E. J., Schulberg, H. C., Parkinson, D. K. and Curtis, E. C. (1991). Factors affecting service utilization for depression in a white collar population. Social Psychiatry and Psychiatric Epidemiology, 26 (5), 230-7. |
| 27 | Dickinson, P., Coggan, C. and Bennett, S. (2003). TRAVELLERS: a school-based early intervention programme helping young people manage and process change, loss and transition. Pilot phase findings. Australian and New Zealand Journal of Psychiatry, 37 (3), 299-306. |
| 28 | Dooley, D. and Catalano, R. (1984). Why the economy predicts help-seeking: a test of competing explanations. Journal of Health and Social Behavior, 25 (2), 160-76. |
| 29 | Drapeau, A., Boyer, R. and Lesage, A. (2009). The influence of social anchorage on the gender difference in the use of mental health services. The Journal of Behavioral Health Services & Research, 36 (3), 372-384. |
| 30 | Edwards, S., Tinning, L., Brown, J. S., Boardman, J. and Weinman, J. (2007). Reluctance to seek help and the perception of anxiety and depression in the United kingdom: a pilot vignette study. The Journal of Nervous and Mental Disease, 195 (3), 258-61. |
| 31 | Farrand, P., Parker, M. and Lee, C. (2007). Intention of adolescents to seek professional help for emotional and behavioural difficulties. Health & Social Care in the Community, 15 (5), 464-473. |
| 32 | Fisher, L. J. and Goldney, R. D. (2003). Differences in community mental health literacy in older and younger Australians. International Journal Geriatric Psychiatry, 18 (1), 33-40. |
| 33 | Fortney, J., Rost, K. and Zhang, M. (1998). A joint choice model of the decision to seek depression treatment and choice of provider sector. Medical Care, 36 (3), 307-20. |
| 34 | Fosu, G. B. (1995). Women's orientation toward help-seeking for mental disorders. Social Science & Medicine, 40 (8), 1029-40. |
| 35 | Gadalla, T. M. (2008). Comparison of users and non-users of mental health services among depressed women: a national study. Women & Health, 47 (1), 1-19. |
| 36 | Galbaud du Fort, G., Newman, S. C., Boothroyd, L. J. and Bland, R. C. (1999). Treatment seeking for depression: role of depressive symptoms and comorbid psychiatric diagnoses. Journal of Affective Disorders, 52 (1-3), 31-40. |
| 37 | Garland, A. F. and Zigler, E. F. (1994). Psychological correlates of help-seeking attitudes among children and adolescents. American Journal of Orthopsychiatry, 64 (4), 586-93. |
| 38 | Gater, R. and Goldberg, D. (1991). Pathways to psychiatric care in South Manchester. British Journal of Psychiatry, 159 90-6. |
| 39 | Golberstein, E., Eisenberg, D. and Gollust, S. E. (2008). Perceived stigma and mental health care seeking. Psychiatric Services, 59 (4), 392-9. |
| 40 | Goldney, R. D., Fisher, L. J., Wilson, D. H. and Cheok, F. (2002). Mental health literacy of those with major depression and suicidal ideation: an impediment to help seeking. Suicide and Life Threatening Behavior, 32 (4), 394-403. |
| 41 | Greenley, J. R., Mechanic, D. and Cleary, P. D. (1987). Seeking help for psychological problems. A replication and extension. Medical Care, 25 (12), 1113-28. |
| 42 | Grella, C. E., Greenwell, L., Mays, V. M. and Cochran, S. D. (2009). Influence of gender, sexual orientation, and need on treatment utilization for substance use and mental disorders: findings from the California Quality of Life Survey. BMC Psychiatry,9 (52), 10.1186/1471-244X-9-52 |
| 43 | Haas, A., Koestner, B., Rosenberg, J., Moore, D., Garlow, S. J., Sedway, J., Nicholas, L., Hendin, H., Mann, J. J. and Nemeroff, C. B. (2008). An interactive web-based method of outreach to college students at risk for suicide. Journal of American College of Health, 57 (1), 15-22. |
| 44 | Hoyt, D. R., Conger, R. D., Valde, J. G. and Weihs, K. (1997). Psychological distress and help seeking in rural America. American Journal of Community Psychology, 25 (4), 449-70. |
| 45 | Hsu, L. and Alden, L. E. (2008). Cultural influences on willingness to seek treatment for social anxiety in Chinese- and European-heritage students. Cultural Diversity and Ethnic Minority Psychology, 14 (3), 215-23. |
| 46 | Huang, Z. J., Wong, F. Y., Ronzio, C. R. and Yu, S. M. (2007). Depressive symptomatology and mental health help-seeking patterns of U.S.- and foreign-born mothers. Maternal and Child Health Journal, 11 (3), 257-67. |
| 47 | Johnson, D. M. and Zlotnick, C. (2007). Utilization of mental health treatment and other services by battered women in shelters. Psychiatric Services, 58 (12), 1595-7. |
| 48 | Jorm, A. F., Christensen, H. and Griffiths, K. M. (2006). The public's ability to recognize mental disorders and their beliefs about treatment: changes in Australia over 8 years. Australian and New Zealand Journal of Psychiatry, 40 (1), 36-41. |
| 49 | Jorm, A. F., Griffiths, K. M., Christensen, H., Korten, A. E., Parslow, R. A. and Rodgers, B. (2003). Providing information about the effectiveness of treatment options to depressed people in the community: a randomized controlled trial of effects on mental health literacy, help-seeking and symptoms. Psychological Medicine, 33 (6), 1071-9. |
| 50 | Jorm, A. F., Kelly, C. M., Wright, A., Parslow, R. A., Harris, M. G. and McGorry, P. D. (2006). Belief in dealing with depression alone: results from community surveys of adolescents and adults. Journal of Affective Disorders, 96 (1-2), 59-65. |
| 51 | Judd, F., Jackson, H., Komiti, A., Murray, G., Fraser, C., Grieve, A. and Gomez, R. (2006). Help-seeking by rural residents for mental health problems: the importance of agrarian values. Australian and New Zealand Journal of Psychiatry, 40 (9), 769-76. |
| 52 | Judd, F., Jackson, H., Komiti, A., Murray, G. and Fraser, C. (2007). Service utilisation by rural residents with mental health problems. Australasian Psychiatry, 15 (3), 185-90. |
| 53 | Katerndahl, D. A. (2002). Factors influencing care seeking for a self-defined worst panic attack. Psychiatric Services, 53 (4), 464-70. |
| 54 | Keefe, S. E. (1982). Help-seeking behavior among foreign-born and native-born Mexican Americans. Social Science & Medicine, 16 (16), 1467-72. |
| 55 | Kendler, K. S. (1995). Is seeking treatment for depression predicted by a history of depression in relatives? Implications for family studies of affective disorder. Psychological Medicine, 25 (4), 807-14. |
| 56 | Kovess-Masfety, V., Saragoussi, D., Sevilla-Dedieu, C., Gilbert, F., Suchocka, A., Arveiller, N., Gasquet, I., Younes, N. and Hardy-Bayle, M. C. (2007). What makes people decide who to turn to when faced with a mental health problem? Results from a French survey. BMC Public Health, 7 188. |
| 57 | Laitinen-Krispijn, S., Van der Ende, J., Wierdsma, A. I. and Verhulst, F. C. (1999). Predicting adolescent mental health service use in a prospective record-linkage study. Journal of the American Academy of Child & Adolescent Psychiatry, 38 (9), 1073-80. |
| 58 | Lam, A. P. and Kavanagh, D. J. (1996). Help seeking by immigrant Indochinese psychiatric patients in Sydney, Australia. Psychiatric Services, 47 (9), 993-5. |
| 59 | Lauber, C., Nordt, C., Falcato, L. and Rossler, W. (2001). Lay recommendations on how to treat mental disorders. Social Psychiatry and Psychiatric Epidemiology, 36 (11), 553-6. |
| 60 | Loewenthal, K. M., Cinnirella, M., Evdoka, G. and Murphy, P. (2001). Faith conquers all? Beliefs about the role of religious factors in coping with depression among different cultural-religious groups in the UK. British Journal of Medical Psychology, 74 (Pt 3), 293-303. |
| 61 | Logsdon, M. C., Usui, W., Pinto-Foltz, M. and Rakestraw, V. L. (2009). Intention to seek depression treatment in adolescent mothers and a comparison group of adolescent girls. Archives of Psychiatric Nursing, 23 (1), 41-9. |
| 62 | Mackenzie, C. S., Gekoski, W. L. and Knox, V. J. (2006). Age, gender, and the underutilization of mental health services: the influence of help-seeking attitudes. Aging & Mental Health, 10 (6), 574-82. |
| 63 | Mackenzie, C. S., Scott, T., Mather, A. and Sareen, J. (2008). Older adults' help-seeking attitudes and treatment beliefs concerning mental health problems. American Journal of Geriatric Psychiatry, 16 (12), 1010-9. |
| 64 | Marcell, A. V. and Halpern-Felsher, B. L. (2005). Adolescents' health beliefs are critical in their intentions to seek physician care. Preventative Medicine, 41 (1), 118-25. |
| 65 | Marks, A., Malizio, J., Hoch, J., Brody, R. and Fisher, M. (1983). Assessment of health needs and willingness to utilize health care resources of adolescents in a suburban population. Journal of Pediatrics, 102 (3), 456-60. |
| 66 | Mo, P. K. H., Mak, W. W. S. and Kwan, C. S. Y. (2007). Cultural Change and Chinese Immigrants' Distress and Help-Seeking in Hong Kong. Journal of Ethnic & Cultural Diversity in Social Work, 15 (3-4), 129-151. |
| 67 | Mojtabai, R., Olfson, M. and Mechanic, D. (2002). Perceived need and help-seeking in adults with mood, anxiety, or substance use disorders. Archives of General Psychiatry, 59 (1), 77-84. |
| 68 | Mojtabai, R. (2007). Americans' attitudes toward mental health treatment seeking: 1990-2003. Psychiatric Services, 58 (5), 642-51. |
| 69 | Narikiyo, T. A. and Kameoka, V. A. (1992). Attributions of mental illness and judgments about help seeking among Japanese-American and White American students. Journal of Counseling Psychology, 39 (3), 363-369. |
| 70 | Neighbors, H. W. (1984). Professional help use among black Americans: implications for unmet need. American Journal of Community Psychology, 12 (5), 551-66. |
| 71 | Neighbors, H. W. (1985). Seeking professional help for personal problems: black Americans' use of health and mental health services. Community Mental Health Journal, 21 (3), 156-66. |
| 72 | Ng, T. P., Jin, A. Z., Ho, R., Chua, H. C., Fones, C. S. and Lim, L. (2008). Health beliefs and help seeking for depressive and anxiety disorders among urban Singaporean adults. Psychiatric Services, 59 (1), 105-8. |
| 73 | Olfson, M. and Klerman, G. L. (1992). Depressive symptoms and mental health service utilization in a community sample. Social Psychiatry and Psychiatric Epidemiology, 27 (4), 161-7. |
| 74 | Oliver, M. I., Pearson, N., Coe, N. and Gunnell, D. (2005). Help-seeking behaviour in men and women with common mental health problems: cross-sectional study. British Journal of Psychiatry, 186 297-301. |
| 75 | Oliver, J. M., Reed, C. K. S., Katz, B. M. and Haugh, J. A. (1999). Students' self-reports of help-seeking: The impact of psychological problems, stress, and demographic variables on utilization of formal and informal support. Social Behavior and Personality, 27 (2), 109-128. |
| 76 | Parker, G., Chan, B. and Tully, L. (2006). Depression and help-seeking in a western sample of 'highly acculturated' Chinese and controls. Journal of Affective Disorders, 94 (1-3), 239-42. |
| 77 | Peifer, K. L., Hu, T. and Vega, W. (2000). Help seeking by persons of Mexican origin with functional impairments. Psychiatric Services, 51 (10), 1293-8. |
| 78 | Perry, B. L., Pescosolido, B. A., Martin, J. K., McLeod, J. D. and Jensen, P. S. (2007). Comparison of public attributions, attitudes, and stigma in regard to depression among children and adults. Psychiatric Services, 58 (5), 632-5. |
| 79 | Phelan, J. C., Yang, L. H. and Cruz-Rojas, R. (2006). Effects of attributing serious mental illnesses to genetic causes on orientations to treatment. Psychiatric Services, 57 (3), 382-7. |
| 80 | Pillay, U. and Rao, K. (2002). The structure and function of social support in relation to help-seeking behavior. Family Therapy, 29 (3), 153-167. |
| 81 | Portes, A., Kyle, D. and Eaton, W. W. (1992). Mental illness and help-seeking behavior among Mariel Cuban and Haitian refugees in south Florida. Journal of Health and Social Behavior, 33 (4), 283-98. |
| 82 | Ramos-Sanchez, L. and Atkinson, D. R. (2009). The relationships between Mexican American acculturation, cultural values, gender, and help-seeking intentions. Journal of Counseling & Development, 87 (1), 62-71. |
| 83 | Razali, S. M. and Najib, M. A. (2000). Help-seeking pathways among Malay psychiatric patients. International Journal of Social Psychiatry, 46 (4), 281-9. |
| 84 | Realmuto, G. M., Bernstein, G. A., Maglothin, M. A. and Pandey, R. S. (1992). Patterns of utilization of outpatient mental health services by children and adolescents. Hospital & Community Psychiatry, 43 (12), 1218-23. |
| 85 | Rew, L., Resnick, M. and Blum, R. (1997). An exploration of help-seeking behaviours in female hispanic adolescents. Family & Community Health, 20 (3), 1-15. |
| 86 | Rickwood, D. J. and Braithwaite, V. A. (1994). Social-psychological factors affecting help-seeking for emotional problems. Social Science & Medicine, 39 (4), 563-72. |
| 87 | Riedel-Heller, S. G., Matschinger, H. and Angermeyer, M. C. (2005). Mental disorders--who and what might help? Help-seeking and treatment preferences of the lay public. Social Psychiatry and Psychiatric Epidemiology, 40 (2), 167-74. |
| 88 | Rose, D., Thornicroft, G., Pinfold, V. and Kassam, A. (2007). 250 labels used to stigmatise people with mental illness. BMC Health Services Research, 7 97. |
| 89 | Russell, V., Gaffney, P., Collins, K., Bergin, A. and Bedford, D. (2004). Problems experienced by young men and attitudes to help-seeking in a rural Irish community. Irish Journal of Psychological Medicine, 21 (1), 6-10. |
| 90 | Santor, D. A., Poulin, C., LeBlanc, J. C. and Kusumakar, V. (2007). Online health promotion, early identification of difficulties, and help seeking in young people. Journal of the American Academy of Child & Adolescent Psychiatry, 46 (1), 50-9. |
| 91 | Saunders, S., Resnick, M., Hoberman, H. and Blum, R. (1994). Formal help-seeking behavior of adolescents identifying themselves as having mental health problems. Journal of the American Academy of Child & Adolescent Psychiatry, 33 (5), 718-28. |
| 92 | Sawyer, M. G., Sarris, A., Baghurst, P. A., Cornish, C. A. and Kalucy, R. S. (1990). The prevalence of emotional and behaviour disorders and patterns of service utilisation in children and adolescents. Australian and New Zealand Journal of Psychiatry, 24 (3), 323-30. |
| 93 | Schomerus, G., Matschinger, H. and Angermeyer, M. C. (2009). The stigma of psychiatric treatment and help-seeking intentions for depression. European Archives of Psychiatry and Clinical Neuroscience, 259 (5), 298-306. |
| 94 | Schonert-Reichl, K. and Muller, J. (1996). Correlates of help-seeking in adolescence. Journal of Youth and Adolescence, 25 (6), 705-731. |
| 95 | Segal, D. L., Coolidge, F. L., Mincic, M. S. and O'Riley, A. (2005). Beliefs about mental illness and willingness to seek help: a cross-sectional study. Aging & Mental Health, 9 (4), 363-7. |
| 96 | Segal, S. P., Hardiman, E. R. and Hodges, J. Q. (2002). Factors in decisions to seek help from self-help and co-located community mental health agencies. American Journal of Orthopsychiatry, 72 (2), 241-9. |
| 97 | Segee, P. F., Maguire, L., Ross, J., Malik, M. L., Colket, J. and Davidson, J. R. (1999). Demographics, treatment seeking, and diagnoses of anxiety support group participants. Journal of Anxiety Disorders, 13 (3), 315-34. |
| 98 | Sen, B. (2004). Adolescent propensity for depressed mood and help seeking: race and gender differences. The Journal of Mental Health Policy and Economics, 7 (3), 133-45. |
| 99 | Shea, M. and Yeh, C. J. (2008). Asian American students' cultural values, stigma, and relational self-construal: Correlates and attitudes toward professional help seeking. Journal of Mental Health Counseling, 30 (2), 157-172. |
| 100 | Sheikh, S. and Furnham, A. (2000). A cross-cultural study of mental health beliefs and attitudes towards seeking professional help. Social Psychiatry and Psychiatric Epidemiology, 35 (7), 326-34. |
| 101 | Smith, J. P., Tran, G. Q. and Thompson, R. D. (2008). Can the theory of planned behavior help explain men's psychological help-seeking? Evidence for a mediation effect and clinical implications. Psychology of Men & Masculinity, 9 (3), 179-192. |
| 102 | Snell, C. L. (1991). Help-seeking behavior among young street males. Smith College Studies in Social Work, 61 293-304. |
| 103 | Steel, Z., McDonald, R., Silove, D., Bauman, A., Sandford, P., Herron, J. and Minas, I. H. (2006). Pathways to the first contact with specialist mental health care. Australian and New Zealand Journal of Psychiatry, 40 (4), 347-54. |
| 104 | Stiffman, A. R., Earls, F., Robins, L. N. and Jung, K. G. (1988). Problems and help seeking in high-risk adolescent patients of health clinics. Journal of Adolescent Health Care, 9 (4), 305-9. |
| 105 | Tijhuis, M. A., Peters, L. and Foets, M. (1990). An orientation toward help-seeking for emotional problems. Social Science & Medicine, 31 (9), 989-995. |
| 106 | Van Voorhees, B. W., Fogel, J., Houston, T. K., Cooper, L. A., Wang, N. Y. and Ford, D. E. (2006). Attitudes and illness factors associated with low perceived need for depression treatment among young adults. Social Psychiatry and Psychiatric Epidemiology, 41 (9), 746-54. |
| 107 | Vanheusden, K., van der Ende, J., Mulder, C. L., van Lenthe, F. J., Verhulst, F. C. and Mackenbach, J. P. (2009). Beliefs about mental health problems and help-seeking behavior in Dutch young adults. Social Psychiatry and Psychiatric Epidemiology, 44 (3), 239-46. |
| 108 | Veroff, J. B. (1981). The dynamics of help-seeking in men and women: a national survey study. Psychiatry, 44 (3), 189-200. |
| 109 | Vogel, D. L., Wade, N. G., Wester, S. R., Larson, L. and Hackler, A. H. (2007). Seeking help from a mental health professional: The influence of one's social network. Journal of Clinical Psychology, 63 (3), 233-245. |
| 110 | Wang, J., Patten, S. B., Williams, J. V., Currie, S., Beck, C. A., Maxwell, C. J. and El-Guebaly, N. (2005). Help-seeking behaviours of individuals with mood disorders. Canadian Journal of Psychiatry, 50 (10), 652-9. |
| 111 | Ward, E. C. and Heidrich, S. M. (2009). African American women's beliefs about mental illness, stigma, and preferred coping behaviors. Research in Nursing & Health, 32 (5), 480-92. |
| 112 | Wilson, C., Deane, F. and Ciarrochi, J. (2005). Can hopelessness and adolescents’ beliefs and attitudes about seeking help account for help negation? Journal of Clinical Psychology, 61 (12), 1525–1539. |
| 113 | Wilson, C., Deane, F., Ciarrochi, J. and Rickwood, D. (2005). Measuring help-seeking intentions: Properties of the General Help-Seeking Questionnaire. Canadian Journal of Counselling, 39 (1), 15-28. |
| 114 | Wilson, C., Deane, F., KL, M. and Dalley, A. (2009). Adolescents’ suicidal thinking and reluctance to consult general medical practitioners. Journal of Youth and Adolescence,15 July 2009 (published online) 10.1007/s10964-009-9436-6 |
| 115 | Wright, A., Jorm, A. F., Harris, M. G. and McGorry, P. D. (2007). What's in a name? Is accurate recognition and labelling of mental disorders by young people associated with better help-seeking and treatment preferences? Social Psychiatry and Psychiatric Epidemiology, 42 (3), 244-50. |
| 116 | Wright, A., McGorry, P. D., Harris, M. G., Jorm, A. F. and Pennell, K. (2006). Development and evaluation of a youth mental health community awareness campaign - The Compass Strategy. BMC Public Health, 6 215. |
| 117 | Wrigley, S., Jackson, H., Judd, F. and Komiti, A. (2005). Role of stigma and attitudes toward help-seeking from a general practitioner for mental health problems in a rural town. Australian and New Zealand Journal of Psychiatry, 39 (6), 514-21. |
| 118 | Wu, P., Hoven, C. W., Cohen, P., Liu, X., Moore, R. E., Tiet, Q., Okezie, N., Wicks, J. and Bird, H. R. (2001). Factors associated with use of mental health services for depression by children and adolescents. Psychiatric Services, 52 (2), 189-95. |
| 119 | Ying, Y. W. and Miller, L. S. (1992). Help-seeking behavior and attitude of Chinese Americans regarding psychological problems. American Journal of Community Psychology, 20 (4), 549-56. |
| 120 | Zachrisson, H. D., Rodje, K. and Mykletun, A. (2006). Utilization of health services in relation to mental health problems in adolescents: a population based survey. BMC Public Health, 6 34. |
| 121 | Zimmerman, F. J. (2005). Social and economic determinants of disparities in professional help-seeking for child mental health problems: evidence from a national sample. Health Services Research, 40 (5 Pt 1), 1514-33. |

**Criterion 2. Not young adults (12-25yrs) (n=32)**

| 1 | Abe-Kim, J., Takeuchi, D. and Hwang, W. C. (2002). Predictors of help seeking for emotional distress among Chinese Americans: family matters. Journal of Consulting and Clinical Psychology, 70 (5), 1186-90. |
| --- | --- |
| 2 | Barney, L. J., Griffiths, K. M., Christensen, H. and Jorm, A. F. (2009). Exploring the nature of stigmatising beliefs about depression and help-seeking: implications for reducing stigma. BMC Public Health, 9 61. |
| 3 | Blumenthal, R. and Endicott, J. (1996). Barriers to seeking treatment for major depression. Depression and Anxiety, 4 (6), 273-8. |
| 4 | Cusack, J., Deane, F., Wilson, C. and Ciarrochi, J. (2004). Who influence men to go to therapy? Reports from men attending psychological services. International Journal for the Advancement of Counselling, 26 (3), 271-283. |
| 5 | Cusack, J., Deane, F., Wilson, C. and Ciarrochi, J. (2006). Emotional expression, perceptions of therapy, and help-seeking intentions in men attending therapy services. Psychology of Men & Masculinity, 7 (2), 69-82. |
| 6 | Duran, B., Oetzel, J., Lucero, J., Jiang, Y., Novins, D. K., Manson, S. and Beals, J. (2005). Obstacles for rural American Indians seeking alcohol, drug, or mental health treatment. Journal of Consulting and Clinical Psychology, 73 (5), 819-29. |
| 7 | Fox, J. C., Blank, M., Berman, J. and Rovnyak, V. G. (1999). Mental disorders and help seeking in a rural impoverished population. International Journal of Psychiatry in Medicine, 29 (2), 181-95. |
| 8 | Fox, J. C., Blank, M., Rovnyak, V. G. and Barnett, R. Y. (2001). Barriers to help seeking for mental disorders in a rural impoverished population. Community Mental Health Journal, 37 (5), 421-36. |
| 9 | Ho, K. P., Hunt, C. and Li, S. (2008). Patterns of help-seeking behavior for anxiety disorders among the Chinese speaking Australian community. Social Psychiatry and Psychiatric Epidemiology, 43 (11), 872-877. |
| 10 | Judd, F., Jackson, H., Fraser, C., Murray, G., Robins, G. and Komiti, A. (2006). Understanding suicide in Australian farmers. Social Psychiatry and Psychiatric Epidemiology, 41 (1), 1-10. |
| 11 | Maguen, S. and Litz, B. T. (2006). Predictors of barriers to mental health treatment for Kosovo and Bosnia peacekeepers: a preliminary report. Military Medicine, 171 (5), 454-8. |
| 12 | McCabe, M. P. and Leas, L. (2008). A qualitative study of primary health care access, barriers and satisfaction among people with mental illness. Psychology, Health & Medicine, 13 (3), 303-12. |
| 13 | Meltzer, H., Bebbington, P., Brugha, T., Farrell, M., Jenkins, R. and Lewis, G. (2000). The reluctance to seek treatment for neurotic disorders. Journal of Mental Health, 9 (3), 319-327. |
| 14 | Mojtabai, R. (2009). Unmet need for treatment of major depression in the United States. Psychiatric Services, 60 (3), 297-305. |
| 15 | Nadeem, E., Lange, J. M., Edge, D., Fongwa, M., Belin, T. and Miranda, J. (2007). Does stigma keep poor young immigrant and U.S.-born Black and Latina women from seeking mental health care? Psychiatric Services, 58 (12), 1547-54. |
| 16 | Olfson, M., Guardino, M., Struening, E., Schneier, F. R., Hellman, F. and Klein, D. F. (2000). Barriers to the treatment of social anxiety. American Journal of Psychiatry, 157 (4), 521-7. |
| 17 | Outram, S., Murphy, B. and Cockburn, J. (2004). Factors associated with accessing professional help for psychological distress in midlife Australian women. Journal of Mental Health Counseling, 13 (2), 185-196. |
| 18 | Pagura, J., Fotti, S., Katz, L. Y. and Sareen, J. (2009). Help seeking and perceived need for mental health care among individuals in Canada with suicidal behaviors. Psychiatric Services, 60 (7), 943-949. |
| 19 | Robb, C., Haley, W. E., Becker, M. A., Polivka, L. A. and Chwa, H. J. (2003). Attitudes towards mental health care in younger and older adults: similarities and differences. Aging & Mental Health, 7 (2), 142-52. |
| 20 | Sareen, J., Jagdeo, A., Cox, B. J., Clara, I., ten Have, M., Belik, S. L., de Graaf, R. and Stein, M. B. (2007). Perceived barriers to mental health service utilization in the United States, Ontario, and the Netherlands. Psychiatric Services, 58 (3), 357-64. |
| 21 | Seedat, S., Stein, D. J., Berk, M. and Wilson, Z. (2002). Barriers to treatment among members of a mental health advocacy group in South Africa. Social Psychiatry and Psychiatric Epidemiology, 37 (10), 483-487. |
| 22 | Shin, J. K. (2002). Help-seeking behaviors by Korean immigrants for depression. Issues in Mental Health Nursing, 23 (5), 461-76. |
| 23 | Stuber, J., Galea, S., Boscarino, J. A. and Schlesinger, M. (2006). Was there unmet mental health need after the September 11, 2001 terrorist attacks? Social Psychiatry and Psychiatric Epidemiology, 41 (3), 230-240. |
| 24 | Sussman, L. K., Robins, L. N. and Earls, F. (1987). Treatment-seeking for depression by black and white Americans. Social Science & Medicine, 24 (3), 187-96. |
| 25 | Svanborg, C., Rosso, M. S., Lutzen, K., Wistedt, A. A. and Baarnhielm, S. (2008). Barriers in the help-seeking process: a multiple-case study of early-onset dysthymia in Sweden. Nordic Journal of Psychiatry, 62 (5), 346-53. |
| 26 | Thompson, A., Hunt, C. and Issakidis, C. (2004). Why wait? Reasons for delay and prompts to seek help for mental health problems in an Australian clinical sample. Social Psychiatry and Psychiatric Epidemiology, 39 (10), 810-817. |
| 27 | Thurston, I. B. and Phares, V. (2008). Mental health service utilization among African American and Caucasian mothers and fathers. Journal of Consulting and Clinical Psychology, 76 (6), 1058-67. |
| 28 | Van Hook, M. P. (1996). Challenges to identifying and treating women with depression in rural primary care. Social Work in Health Care, 23 (3), 73-92. |
| 29 | Van Hook, M. P. (1999). Women's help-seeking patterns for depression. Social Work in Health Care, 29 (1), 15-34. |
| 30 | Wang, P. S., Gruber, M. J., Powers, R. E., Schoenbaum, M., Speier, A. H., Wells, K. B. and Kessler, R. C. (2007). Mental health service use among hurricane Katrina survivors in the eight months after the disaster. Psychiatric Services, 58 (11), 1403-1411. |
| 31 | Wu, M. C., Kviz, F. J. and Miller, A. M. (2009). Identifying individual and contextual barriers to seeking mental health services among Korean American immigrant women. Issues in Mental Health Nursing, 30 (2), 78-85. |
| 32 | Wynaden, D., Chapman, R., Orb, A., McGowan, S., Zeeman, Z. and Yeak, S. (2005). Factors that influence Asian communities' access to mental health care. International Journal of Mental Health Nursing, 14 (2), 88-95. |

**Criterion 3. Review (n=11)**

| 1 | Bristow, K. and Patten, S. (2002). Treatment-seeking rates and associated mediating factors among individuals with depression. Canadian Journal of Psychiatry, 47 (7), 660-5. |
| --- | --- |
| 2 | Dennis, C. L. and Chung-Lee, L. (2006). Postpartum depression help-seeking barriers and maternal treatment preferences: a qualitative systematic review. Birth, 33 (4), 323-31. |
| 3 | Haynes, N. M. (2002). Addressing students' social and emotional needs: the role of mental health teams in schools. Journal of Health & Social Policy, 16 (1-2), 109-23. |
| 4 | Hodges, C. A., O'Brien, M. S. and McGorry, P. D. (2007). headspace: National Youth Mental Health Foundation: making headway with rural young people and their mental health. Australian Journal of Rural Health, 15 (2), 77-80. |
| 5 | Lincoln, C. V. and McGorry, P. (1995). Who cares? Pathways to psychiatric care for young people experiencing a first episode of psychosis. Psychiatric Services, 46 (11), 1166-71. |
| 6 | Rickwood, D., Deane, F. and Wilson, C. (2007). When and how do young people seek professional help for mental health problems? Medical Journal of Australia, 187 (7), S35-S39. |
| 7 | Rickwood, D., Deane, F. P., Wilson, C. J. and Ciarrochi, J. (2005). Young people's help-seeking for mental health problems. Australian e-Journal for the Advancement of Mental Health, 4 (3 Supplement), 1-34. |
| 8 | Rogler, L. H. and Cortes, D. E. (1993). Help-seeking pathways: a unifying concept in mental health care. American Journal of Psychiatry, 150 (4), 554-61. |
| 9 | Smith, L. (1992). Help seeking in alcohol-dependent females. Alcohol and Alcoholism, 27 (1), 3-9. |
| 10 | Srebnik, D., Cauce, A. and Baydar, N. (1996). Help-Seeking Pathways for Children and Adolescents. Journal of Emotional and Behavioral Disorders, 4 (4), 210-220. |
| 11 | Zwaanswijk, M., Verhaak, P. F., Bensing, J. M., van der Ende, J. and Verhulst, F. C. (2003). Help seeking for emotional and behavioural problems in children and adolescents: a review of recent literature. European Child & Adolescent Psychiatry, 12 (4), 153-61. |

**Criterion 4. Not from the general community (n=11)**

*Prison Study (n=7)*

| 1 | Abram, K. M., Paskar, L. D., Washburn, J. J. and Teplin, L. A. (2008). Perceived barriers to mental health services among youths in detention. Journal of the American Academy of Child & Adolescent Psychiatry, 47 (3), 301-8. |
| --- | --- |
| 2 | Deane, F., Skogstad, P. and Williams, M. (1999). Impact of attitudes, ethnicity and quality of prior therapy on New Zealand male prisoners’ intentions to seek professional psychological help. International Journal for the Advancement of Counselling, 21 (1), 55-67. |
| 3 | Howerton, A., Byng, R., Campbell, J., Hess, D., Owens, C. and Aitken, P. (2007). Understanding help seeking behaviour among male offenders: qualitative interview study. BMJ. British Medical Journal, 334 (7588), 303. |
| 4 | Morgan, R. D., Rozycki, A. T. and Wilson, S. (2004). Inmate Perceptions of Mental Health Services. Professional Psychology, Research and Practice, 35 (4), 389-396. |
| 5 | Skogstad, P., Deane, F. P. and Spicer, J. (2006). Social-cognitive determinants of help-seeking for mental health problems among prison inmates. Criminal Behaviour and Mental Health, 16 (1), 43-59. |
| 6 | Skogstad, P., Deane, F. P. and Spicer, J. (2006). Barriers to Help-seeking Among New Zealand Prison Inmates. Journal of Offender Rehabilitation, 42 (2), 1-24. |
| 7 | Williams, M. W., Skogstad, P. and Deane, F. P. (2002). Attitudes of male prisoners toward seeking professional psychological help. Journal of Offender Rehabilitation, 34 (2), 49-61. |

*Military study (n=4)*

| 1 | Eaton, K. M., Hoge, C. W., Messer, S. C., Whitt, A. A., Cabrera, O. A., McGurk, D., Cox, A. and Castro, C. A. (2008). Prevalence of mental health problems, treatment need, and barriers to care among primary care-seeking spouses of military service members involved in Iraq and Afghanistan deployments. Military Medicine, 173 (11), 1051-6. |
| --- | --- |
| 2 | Snell, F. and Tusaie, K. R. (2008). Veterans reported reasons for seeking mental health treatment. Archives of Psychiatric Nursing, 22 (5), 313-314. |
| 3 | Stecker, T., Fortney, J. C., Hamilton, F. and Ajzen, I. (2007). An assessment of beliefs about mental health care among veterans who served in Iraq. Psychiatric Services, 58 (10), 1358-1361. |
| 4 | Warner, C. H., Appenzeller, G. N., Mullen, K., Warner, C. M. and Grieger, T. (2008). Soldier attitudes toward mental health screening and seeking care upon return from combat. Military Medicine, 173 (6), 563-569. |

**Criterion 5. Parent or carer study (n=5)**

| 1 | Kerkorian, D., McKay, M. and Bannon, W. M., Jr. (2006). Seeking help a second time: parents'/caregivers' characterizations of previous experiences with mental health services for their children and perceptions of barriers to future use. American Journal of Orthopsychiatry, 76 (2), 161-6. |
| --- | --- |
| 2 | McKay, M. M., McCadam, K. and Gonzales, J. J. (1996). Addressing the barriers to mental health services for inner city children and their caretakers. Community Mental Health Journal, 32 (4), 353-61. |
| 3 | Moskos, M. A., Olson, L., Halbern, S. R. and Gray, D. (2007). Utah youth suicide study: barriers to mental health treatment for adolescents. Suicide and Life Threatening Behavior, 37 (2), 179-186. |
| 4 | Owens, C., Lambert, H., Donovan, J. and Lloyd, K. R. (2005). A qualitative study of help seeking and primary care consultation prior to suicide. British Journal of General Practice, 55 (516), 503-9. |
| 5 | Shanley, D. C., Reid, G. J. and Evans, B. (2008). How parents seek help for children with mental health problems. Administration and Policy in Mental Health, 35 (3), 135-46. |

**Criterion 6. Data not extractable (n=2)**

| 1 | Barker, L. A. and Adelman, H. S. (1994). Mental health and help-seeking among ethnic minority adolescents. Journal of Adolescence, 17 (3), 251-263. |
| --- | --- |
| 2 | Luu, T. D., Leung, P. and Nash, S. G. (2009). Help-seeking attitudes among Vietnamese Americans: The impact of acculturation, cultural barriers, and spiritual beliefs. Social Work in Mental Health, 7 (5), 476-493. |

**Criterion 7. Not for depression or anxiety (n=86 )**

*Drug and alcohol studies (n=44)*

| 1 | Allan, C. (1987). Seeking help for drinking problems from a community-based voluntary agency. Patterns of compliance amongst men and women. British Journal of Addiction, 82 (10), 1143-7. |
| --- | --- |
| 2 | Ballon, B., Kirst, M. and Smith, P. (2004). Youth help-seeking expectancies and their relation to help-seeking behaviours for substance use problems. Addiction Research & Theory, 12 (3), 241-260. |
| 3 | Bennett, J. B. and Lehman, W. E. (2001). Workplace substance abuse prevention and help seeking: comparing team-oriented and informational training. Journal of Occupational Health Psychology, 6 (3), 243-54. |
| 4 | Cahill, M. A., Adinoff, B., Hosig, H., Muller, K. and Pulliam, C. (2003). Motivation for treatment preceding and following a substance abuse program. Addictive Behaviors, 28 (1), 67-79. |
| 5 | Copeland, J., Rees, V. and Swift, W. (1999). Help seeking among a sample entering treatment for cannabis dependence. Australian Family Physician, 28 (6), 540-1. |
| 6 | Copeland, J. (1997). A qualitative study of barriers to formal treatment among women who self-managed change in addictive behaviours. Journal of Substance Abuse Treatment, 14 (2), 183-90. |
| 7 | Cunningham, J. A. and Breslin, F. C. (2004). Only one in three people with alcohol abuse or dependence ever seek treatment. Addictive Behaviors, 29 (1), 221-3. |
| 8 | Cunningham, J. A., Sobell, L. C., Sobell, M. B. and Gaskin, J. (1994). Alcohol and drug abusers' reasons for seeking treatment. Addictive Behaviors, 19 (6), 691-6. |
| 9 | D'Amico E, J. (2005). Factors that impact adolescents' intentions to utilize alcohol-related prevention services. Journal of Behavioral Health Services and Research, 32 (3), 332-40. |
| 10 | Delaney, W., Grube, J. W. and Ames, G. M. (1998). Predicting likelihood of seeking help through the employee assistance program among salaried and union hourly employees. Addiction, 93 (3), 399-410. |
| 11 | Fontanella, B. J. and Turato, E. R. (2005). Spontaneous harm reduction: a barrier for substance-dependent individuals seeking treatment? Revista Brasileira de Psiquiatria, 27 (4), 272-7. |
| 12 | Freyer, J., Tonigan, J. S., Keller, S., Rumpf, H. J., John, U. and Hapke, U. (2005). Readiness for change and readiness for help-seeking: a composite assessment of client motivation. Alcohol and Alcoholism, 40 (6), 540-4. |
| 13 | Freyer-Adam, J., Coder, B., Bischof, G., Baumeister, S. E., Rumpf, H. J., John, U. and Hapke, U. (2008). Predicting utilization of formal and informal help among general hospital inpatients with alcohol use disorders. International Journal of Methods Psychiatric Research, 17 Suppl 1 S70-3. |
| 14 | Grant, B. F. (1997). Barriers to alcoholism treatment: reasons for not seeking treatment in a general population sample. Journal of Studies on Alcohol, 58 (4), 365-71. |
| 15 | Grothues, J. M., Bischof, G., Reinhardt, S., Meyer, C., John, U. and Rumpf, H. J. (2008). Differences in help seeking rates after brief intervention for alcohol use disorders in general practice patients with and without comorbid anxiety or depressive disorders. International Journal of Methods Psychiatric Research, 17 Suppl 1 S74-7. |
| 16 | Hughes, J. R., Cohen, B. and Callas, P. W. (2009). Treatment seeking for smoking cessation among young adults. Journal of Substance Abuse Treatment, 37 (2), 211-3. |
| 17 | Kessler, R. C., Aguilar-Gaxiola, S., Berglund, P. A., Caraveo-Anduaga, J. J., DeWit, D. J., Greenfield, S. F., Kolody, B., Olfson, M. and Vega, W. A. (2001). Patterns and predictors of treatment seeking after onset of a substance use disorder. Archives of General Psychiatry, 58 (11), 1065-71. |
| 18 | Lundgren, L. M., Amaro, H. and Ben-Ami, L. (2005). Factors Associated with Drug Treatment Entry Patterns Among Hispanic Women Injection Drug Users Seeking Treatment. In L. K. H. M.R. De La Rosa, & S.L. Ashenberg Straussner (Ed.), Substance abusing Latinos: Current research on epidemiology, prevention, and treatment. |
| 19 | McCoy, C. B., Metsch, L. R., Chitwood, D. D. and Miles, C. (2001). Drug use and barriers to use of health care services. Substance Use and Misuse, 36 (6-7), 789-806. |
| 20 | Montoya, I. D., Haertzen, C., Hess, J. M., Covi, L., Fudala, P. J., Johnson, R. E. and Gorelick, D. A. (1995). Comparison of psychological symptoms between drug abusers seeking and not seeking treatment. The Journal of Nervous and Mental Disease, 183 (1), 50-3. |
| 21 | Neale, J., Sheard, L. and Tompkins, C. N. (2007). Factors that help injecting drug users to access and benefit from services: A qualitative study. Substance Abuse Treatment, Prevention and Policy, 2 31. |
| 22 | Oppenheimer, E., Sheehan, M. and Taylor, C. (1988). Letting the client speak: drug misusers and the process of help seeking. British Journal of Addiction, 83 (6), 635-47. |
| 23 | Perron, B. E., Mowbray, O. P., Glass, J. E., Delva, J., Vaughn, M. G. and Howard, M. O. (2009). Differences in service utilization and barriers among Blacks, Hispanics, and Whites with drug use disorders. Substance Abuse Treatment, Prevention and Policy,4 (3), 10.1186/1747-597x-4-3 |
| 24 | Power, R., Hartnoll, R. and Chalmers, C. (1992). Help-seeking among illicit drug users: some differences between a treatment and nontreatment sample. International Journal of the Addictions, 27 (8), 887-904. |
| 25 | Proudfoot, H. and Teesson, M. (2002). Who seeks treatment for alcohol dependence? Findings from the Australian National Survey of Mental Health and Wellbeing. Social Psychiatry and Psychiatric Epidemiology, 37 (10), 451-6. |
| 26 | Rounsaville, B. J. and Kleber, H. D. (1985). Untreated opiate addicts. How do they differ from those seeking treatment? Archives of General Psychiatry, 42 (11), 1072-7. |
| 27 | Saunders, S. M., Zygowicz, K. M. and D'Angelo, B. R. (2006). Person-related and treatment-related barriers to alcohol treatment. Journal of Substance Abuse Treatment, 30 (3), 261-70. |
| 28 | Sexton, R. L., Carlson, R. G., Leukefeld, C. G. and Booth, B. M. (2008). Barriers to formal drug abuse treatment in the rural south: a preliminary ethnographic assessment. Journal of Psychoactive Drugs, 40 (2), 121-9. |
| 29 | Siebert, D. C. (2005). Help seeking for AOD misuse among social workers: patterns, barriers, and implications. Social Work, 50 (1), 65-75. |
| 30 | Siliquini, R., Morra, A., Versino, E. and Renga, G. (2005). Recreational drug consumers: who seeks treatment? European Journal of Public Health, 15 (6), 580-6. |
| 31 | Spence, R., Wallisch, L. and Smith, S. (2007). Treatment seeking in populations in urban and rural settings on the border. Alcoholism, Clinical and Experimental Research, 31 (6), 1002-11. |
| 32 | Subramaniam, G. A. and Stitzer, M. A. (2009). Clinical characteristics of treatment-seeking prescription opioid vs. heroin-using adolescents with opioid use disorder. Drug and Alcohol Dependence, 101 (1-2), 13-9. |
| 33 | Subramaniam, G. A., Stitzer, M. L., Woody, G., Fishman, M. J. and Kolodner, K. (2009). Clinical characteristics of treatment-seeking adolescents with opioid versus cannabis/alcohol use disorders. Drug and Alcohol Dependence, 99 (1-3), 141-9. |
| 34 | True, W. R., Heath, A. C., Bucholz, K., Slutske, W., Romeis, J. C., Scherrer, J. F., Lin, N., Eisen, S. A., Goldberg, J., Lyons, M. J. and Tsuang, M. T. (1996). Models of treatment seeking for alcoholism: the role of genes and environment. Alcoholism, Clinical and Experimental Research, 20 (9), 1577-81. |
| 35 | Tucker, J. A. and Gladsjo, J. A. (1993). Help-seeking and recovery by problem drinkers: characteristics of drinkers who attended Alcoholics Anonymous or formal treatment or who recovered without assistance. Addictive Behaviors, 18 (5), 529-42. |
| 36 | Tucker, J. A. (1995). Predictors of help-seeking and the temporal relationship of help to recovery among treated and untreated recovered problem drinkers. Addiction, 90 (6), 805-9. |
| 37 | Varney, S. M., Rohsenow, D. J., Dey, A. N., Myers, M. G., Zwick, W. R. and Monti, P. M. (1995). Factors associated with help seeking and perceived dependence among cocaine users. The American Journal of Drug and Alcohol Abuse, 21 (1), 81-91. |
| 38 | Warner, B. D. and Leukefeld, C. G. (2001). Rural-urban differences in substance use and treatment utilization among prisoners. The American Journal of Drug and Alcohol Abuse, 27 (2), 265-80. |
| 39 | Wells, J. E., Horwood, L. J. and Fergusson, D. M. (2007). Reasons why young adults do or do not seek help for alcohol problems. Australian and New Zealand Journal of Psychiatry, 41 (12), 1005-12. |
| 40 | Windle, M., Miller-Tutzauer, C., Barnes, G. M. and Welte, J. (1991). Adolescent perceptions of help-seeking resources for substance abuse. Child Development, 62 (1), 179-89. |
| 41 | Woodward, A. T., Taylor, R. J., Bullard, K. M., Neighbors, H. W., Chatters, L. M. and Jackson, J. S. (2008). Use of professional and informal support by African Americans and Caribbean Blacks with mental disorders. Psychiatric Services, 59 (11), 1292-1298. |
| 42 | Wu, L. T., Ringwalt, C. L. and Williams, C. E. (2003). Use of substance abuse treatment services by persons with mental health and substance use problems. Psychiatric Services, 54 (3), 363-9. |
| 43 | Yen, C. N., Tang, T. C., Liu, S. C., Liu, B. Y., Chang, H. T. and Yen, C. F. (2008). Factors related to abstinence from drug use and seeking help from medical services in Taiwanese heroin and methamphetamine users. The Kaohsiung Journal of Medical Science, 24 (2), 63-71. |
| 44 | Yu, J., Evans, P. C. and Perfetti, L. (2003). Attitudes toward seeking treatment among alcohol-using college students. The American Journal of Drug and Alcohol Abuse, 29 (3), 671-90. |

*Pre or postnatal (n=11)*

| 1 | Abrams, L. S., Dornig, K. and Curran, L. (2009). Barriers to service use for postpartum depression symptoms among low-income ethnic minority mothers in the United States. Qualitative Health Research, 19 (4), 535-51. |
| --- | --- |
| 2 | Friedman, S. H., Heneghan, A. and Rosenthal, M. (2009). Characteristics of women who do not seek prenatal care and implications for prevention. Journal of Obstetric, Gynecologic, and Neonatal Nursing, 38 (2), 174-81. |
| 3 | Goodman, J. H. (2009). Women's attitudes, preferences, and perceived barriers to treatment for perinatal depression. Birth, 36 (1), 60-9. |
| 4 | Holopainen, D. (2002). The experience of seeking help for postnatal depression. Australian Journal of Advanced Nursing, 19 (3), 39-44. |
| 5 | Jesse, D. E., Dolbier, C. L. and Blanchard, A. (2008). Barriers to seeking help and treatment suggestions for prenatal depressive symptoms: Focus groups with rural low-income women. Issues in Mental Health Nursing, 29 (1), 3-19. |
| 6 | Kopelman, R. C., Moel, J., Mertens, C., Stuart, S., Arndt, S. and O'Hara, M. W. (2008). Barriers to care for antenatal depression. Psychiatric Services, 59 (4), 429-432. |
| 8 | Lau, Y. and Wong, D. F. (2008). Are concern for face and willingness to seek help correlated to early postnatal depressive symptoms among Hong Kong Chinese women? A cross-sectional questionnaire survey. International Journal of Nursing Studies, 45 (1), 51-64. |
| 7 | McCarthy, M. and McMahon, C. (2008). Acceptance and experience of treatment for postnatal depression in a community mental health setting. Health Care for Women International, 29 (6), 618-637. |
| 9 | McGarry, J., Kim, H., Sheng, X., Egger, M. and Baksh, L. (2009). Postpartum depression and help-seeking behavior. Journal of Midwifery and Women's Health, 54 (1), 50-6. |
| 10 | Sword, W., Busser, D., Ganann, R., McMillan, T. and Swinton, M. (2008). Women's care-seeking experiences after referral for postpartum depression. Qualitative Health Research, 18 (9), 1161-73. |
| 11 | Woolhouse, H., Brown, S., Krastev, A., Perlen, S. and Gunn, J. (2009). Seeking help for anxiety and depression after childbirth: results of the Maternal Health Study. Archives of Women's Mental Health, 12 (2), 75-83. |

*Psychotic disorders or schizophrenia (n=11)*

| 1 | Bechard-Evans, L., Schmitz, N., Abadi, S., Joober, R., King, S. and Malla, A. (2007). Determinants of help-seeking and system related components of delay in the treatment of first-episode psychosis. Schizophrenia Research, 96 (1-3), 206-14. |
| --- | --- |
| 2 | Boydell, K. M., Gladstone, B. M. and Volpe, T. (2006). Understanding help seeking delay in the prodrome to first episode psychosis: a secondary analysis of the perspectives of young people. Psychiatric Rehabilitation Journal, 30 (1), 54-60. |
| 3 | Coton, X., Poly, S., Hoyois, P., Sophal, C. and Dubois, V. (2008). The healthcare-seeking behaviour of schizophrenic patients in Cambodia. International Journal of Social Psychiatry, 54 (4), 328-337. |
| 4 | Judge, A. M., Perkins, D. O., Nieri, J. and Penn, D. L. (2005). Pathways to care in first episode psychosis: A pilot study on help-seeking precipitants and barriers to care. Journal of Mental Health Counseling, 14 (5), 465-470. |
| 5 | McCann, T. V. and Clark, E. (2003). A grounded theory study of the role that nurses play in increasing clients' willingness to access community mental health services. International Journal of Mental Health Nursing, 12 (4), 279-87. |
| 6 | Morgan, C., Mallett, R., Hutchinson, G., Bagalkote, H., Morgan, K., Fearon, P., Dazzan, P., Boydell, J., McKenzie, K., Harrison, G., Murray, R., Jones, P., Craig, T. and Leff, J. (2005). Pathways to care and ethnicity. 2: Source of referral and help-seeking. Report from the AESOP study. British Journal of Psychiatry, 186 290-6. |
| 7 | Platz, C., Umbricht, D. S., Cattapan-Ludewig, K., Dvorsky, D., Arbach, D., Brenner, H. D. and Simon, A. E. (2006). Help-seeking pathways in early psychosis. Social Psychiatry and Psychiatric Epidemiology, 41 (12), 967-74. |
| 8 | Skeate, A., Jackson, C., Birchwood, M. and Jones, C. (2002). Duration of untreated psychosis and pathways to care in first-episode psychosis. Investigation of help-seeking behaviour in primary care. British Journal of Psychiatry. Supplement, 43 s73-7. |
| 9 | Swartz, M. S., Swanson, J. W. and Hannon, M. J. (2003). Does Fear of Coercion Keep People Away from Mental Health Treatment? Evidence from a Survey of Persons with Schizophrenia and Mental Health Professionals. Behavioral Sciences & the Law, 21 (4), 459-472. |
| 10 | Tang, Y. L., Sevigny, R., Mao, P. X., Jiang, F. and Cai, Z. (2007). Help-seeking behaviors of Chinese patients with schizophrenia admitted to a psychiatric hospital. Administration and Policy in Mental Health, 34 (2), 101-7. |
| 11 | Wong, D. E. (2007). Crucial individuals in the help-seeking pathway of Chinese caregivers of relatives with early psychosis in Hong Kong. Social Work, 52 (2), 127-135. |

*Eating Disorders (n=6)*

| 1 | Cachelin, F. M., Rebeck, R., Veisel, C. and Striegel-Moore, R. H. (2001). Barriers to treatment for eating disorders among ethnically diverse women. International Journal of Eating Disorders, 30 (3), 269-78. |
| --- | --- |
| 2 | Cachelin, F. M. and Striegel-Moore, R. H. (2006). Help seeking and barriers to treatment in a community sample of Mexican American and European American women with eating disorders. International Journal of Eating Disorders, 39 (2), 154-61. |
| 3 | Goodwin, R. D. and Fitzgibbon, M. L. (2002). Social anxiety as a barrier to treatment for eating disorders. International Journal of Eating Disorders, 32 (1), 103-6. |
| 4 | Hepworth, N. and Paxton, S. J. (2007). Pathways to help-seeking in bulimia nervosa and binge eating problems: a concept mapping approach. International Journal of Eating Disorders, 40 (6), 493-504. |
| 5 | Perkins, P. S., Klump, K. L., Iacono, W. G. and McGue, M. (2005). Personality traits in women with anorexia nervosa: evidence for a treatment-seeking bias? International Journal of Eating Disorders, 37 (1), 32-7. |
| 6 | Prouty, A. M., Protinsky, H. O. and Canady, D. (2002). College women: eating behaviors and help-seeking preferences. Adolescence, 37 (146), 353-63. |

*Gambling (n=4)*

| 1 | Evans, L. and Delfabbro, P. H. (2005). Motivators for change and barriers to help-seeking in Australian problem gamblers. Journal of Gambling Studies, 21 (2), 133-55. |
| --- | --- |
| 2 | Hardoon, K., Derevensky, J. L. and Gupta, R. (2003). Empirical measures vs. perceived gambling severity among youth: why adolescent problem gamblers fail to seek treatment. Addictive Behaviors, 28 (5), 933-46. |
| 3 | Pulford, J., Bellringer, M., Abbott, M., Clarke, D., Hodgins, D. and Williams, J. (2009). Reasons for seeking help for a gambling problem: the experiences of gamblers who have sought specialist assistance and the perceptions of those who have not. Journal of Gambling Studies, 25 (1), 19-32. |
| 4 | Pulford, J., Bellringer, M., Abbott, M., Clarke, D., Hodgins, D. and Williams, J. (2009). Barriers to help-seeking for a gambling problem: the experiences of gamblers who have sought specialist assistance and the perceptions of those who have not. Journal of Gambling Studies, 25 (1), 33-48. |

*OCD (n=3)*

| 1 | Belloch, A., Del Valle, G., Morillo, C., Carrio, C. and Cabedo, E. (2009). To seek advice or not to seek advice about the problem: the help-seeking dilemma for obsessive-compulsive disorder. Social Psychiatry and Psychiatric Epidemiology, 44 (4), 257-264. |
| --- | --- |
| 2 | Goodwin, R., Koenen, K. C., Hellman, F., Guardino, M. and Struening, E. (2002). Helpseeking and access to mental health treatment for obsessive-compulsive disorder. Acta Psychiatrica Scandinavica, 106 (2), 143-9. |
| 3 | Mayerovitch, J. I., du Fort, G. G., Kakuma, R., Bland, R. C., Newman, S. C. and Pinard, G. (2003). Treatment seeking for obsessive-compulsive disorder: role of obsessive-compulsive disorder symptoms and comorbid psychiatric diagnoses. Comprehensive Psychiatry, 44 (2), 162-8. |

*Comorbidity or general health care (n=3)*

| 1 | Hahm, H. C. and Segal, S. P. (2005). Failure to seek health care among the mentally ill. American Journal of Orthopsychiatry, 75 (1), 54-62. |
| --- | --- |
| 2 | Johnson, S. and Dwyer, A. (2008). Patient perceived barriers to treatment of depression and anxiety in hemodialysis patients. Clinical Nephrology, 69 (3), 201-6. |
| 3 | Snell, C. L. (2002). Help-seeking and risk-taking behavior among Black street youth: implications for HIV/AIDS prevention and social policy. Journal of Health & Social Policy, 16 (1-2), 21-32. |

*Insomnia (n=1)*

| 1 | Stinson, K., Tang, N. K. and Harvey, A. G. (2006). Barriers to treatment seeking in primary insomnia in the United Kingdom: a cross-sectional perspective. Sleep, 29 (12), 1643-6. |
| --- | --- |

*Dementia of the Alzheimer’s type (n=1)*

| 1 | Blay, S. L., Furtado, A. and Peluso, E. T. (2008). Knowledge and beliefs about help-seeking behavior and helpfulness of interventions for Alzheimer's disease. Aging & Mental Health, 12 (5), 577-86. |
| --- | --- |

*Orofacial Injury (n=1)*

| 1 | Chandra, A., Marshall, G. N., Shetty, V., Paddock, S. M., Wong, E. C., Zatzick, D., Luo, G. and Yamashita, D. D. (2008). Barriers to seeking mental health care after treatment for orofacial injury at a large, urban medical center: concordance of patient and provider perspectives. The Journal of Trauma, 65 (1), 196-202. |
| --- | --- |

*Sexual dysfunction (n=1)*

| 1 | Feldhaus-Dahir, M. (2009). Female sexual dysfunction: barriers to treatment. Urologic Nursing, 29 (2), 81-5; quiz 86. |
| --- | --- |

*Intimate partner violence (n=1)*

| 1 | Prospero, M., & Vohra-Gupta, S. (2008). The use of mental health services among victims of partner violence on college campuses: *Journal of Aggression, Maltreatment & Trauma*, 16(4), 376-390. |
| --- | --- |

*Externalising disorders (n=1)*

| 1 | Vanheusden, K., Mulder, C. L., van der Ende, J., van Lenthe, F. J., Mackenbach, J. P., & Verhulst, F. C. (2008). Young adults face major barriers to seeking help from mental health services. *Patient Education and Counseling, 73*(1), 97-104. |
| --- | --- |
