# Supplementary material for: Perceived barriers and facilitators to mental health help-seeking in young people: a systematic review
Source: BMC Psychiatry. 2010 Dec 30;10:113. doi: 10.1186/1471-244X-10-113 (PMC3022639; doi:10.1186/1471-244X-10-113)
Supplement: Additional file 3 — Qualitative studies included in the review. [file 1471-244X-10-113-S3.DOC]

**Qualitative studies included in the review**

**Table 3.1 -** Included qualitative studies on barriers and facilitators (n=15)

| **#** | **Author** | **Year** | **Loc** | **Age** | **Population** | **Universal** | **N** | **Sex** | **Setting** | **Method** | **Barrier themes reported** | **Facilitator themes reported** |
| --- | --- | --- | --- | --- | --- | --- | --- | --- | --- | --- | --- | --- |
| **1** | **Wilson [39]** | **2007** | AUS | 11-17 | Public high school students | Some -Depression | 359 | M/F | High school | Survey | 1. Embarrassed 2. Did not want to talk to someone who was a stranger 3. Would not want to 4. Prefer a friend or family member 5. Confidentiality or could not be trusted 6. Could not effectively help 7. Rely on oneself rather than seek help 8. Afraid 9. Too busy/No time to seek help 10. Problems did not warrant professional intervention |  |
| **2** | **Timlin-Scalera [36]** | **2003** | USA | 14-18 | Caucasian male high school students | Yes | 26 | M | High school | Interview | 1. Lack of awareness and understanding of services 2. Stigma of being weak and troubled 3. Confidentiality or that others would find out 4. Perceptions of mental health staff as being inaccessible, unavailable and unfamiliar 5. Trust 6. Did not want to burden someone else with their problems 7. Lack of insight into their own problems and need for services 8. Did not want others to take care of them | 1. Someone suggesting it 2. Having previous positive encounters with the mental health field 3. A culture of openness in their homes 4. A perception of assured confidentiality 5. Having what they perceived to be as a very serious problem |
| **3** | **Francis [28]** | **2006** | AUS | Year 9 and 10 | Rural high school students | Yes | 52 | M/F | High school | Focus groups | 1. Lack of specialist local services 2. Lack of transport to services located out of town 3. Exclusionary social practices 4. Fear of social stigma 5. Lack of anonymity 6. Public stigma 7. Self-stigma 8. Culture of self-reliance |  |
| **4** | **Wilson [40]** | **2001** | AUS | 14-17 | Adolescent school students | Yes | 23 | M/F | High school | Focus groups | 1. A lack of education | 1. A strong and open relationship with a potential help-giver 2. Primarily a matter of trust 3. Positive attitudes towards seeking help 4. Knowledge from prior help 5. Knowledge from peer discussion 6. Problem normalisation 7. Successful prior help-seeking |
| **5** | **Lindsey [33]** | **1998** | USA | Year 9 | White high school students | Yes | 41 | M/F | High school | Focus groups | 1. Active negativity 2. Breach of confidentiality 3. Dual roles 4. Judgmental/shows favouritism 5. Non-helpful responses 6. Out of touch with teens 7. Psychologically inaccessible 8. Too busy | 1. Active problem solving 2. Effective listening 3. Empathic 4. Familiarity 5. Genuine 6. Knowledgeable 7. Makes self available 8. Non-judgmental 9. Projects a professional image 10. Relates to teens 11. Trustworthy |
| **6** | **Helms [30]** | **2003** | USA | Year 12 | Year 12 Students | Yes | 32 | M/F | High school | Focus groups | 1. Dual roles: Hard to talk to somebody when you think of them as an enforcer of the school rules 2. Psychologically inaccessible: Never assure you that you can come and talk to them 3. Non-helpful responses: They blow it out of proportion-exaggerate 4. Judgmental/shows favouritism: Some adults don’t see both sides 5. Breach of confidentiality: Not enough privacy in school 6. Out of touch with teens: They don’t know about gangs and drugs 7. Active negativity: Rude and smart aleck 8. Too busy: They have too many kids to deal with |  |
| **7** | **Wisdom [41]** | **2006** | USA | 14-19 | Teenagers with depression (majority) | No – Most had depression | 22 | M/F | High School/  Community | Focus Groups/  Interview | 1. Desire to be normal, e.g., reject diagnosis, normalise symptoms 2. A lack of connection with the provider, e.g., they were not competent, do not listen 3. Desire to be autonomous, e.g., not having a voice in treatment, getting little information about what was happening |  |
| **8** | **Lindsey [34]** | **2006** | USA | 14-18 | At risk African American adolescent boys | No -Depression | 18 | M | Community | Interview | 1. Stigma – shame, embarrassment and exclusion 2. Handle problem on own 3. Pride 4. Expression of emotions viewed as sign of weakness 5. Race of the provider being different to their own | 1. How well the provider treated and engaged them |
| **9** | **Jorm [31]** | **2007** | AUS | 12-25 | Young people | Yes | 3746 | M/F | Community | Survey | Participants given list of sources of help and asked: what might stop you from seeking help from this (person/service)? Professional sources and those scoring more than 5% only are listed.  ***Percentage of participants endorsing barrier (%) by source type:***  *General practitioner*   1. None (23%) 2. Too embarrassed/shy (18%) 3. Concern doctor might feel negatively about you (10%) 4. Negative feelings/self/perceptions (7%) 5. Denial/pride (5%)   *Counsellor*   1. Too embarrassed/shy (26%) 2. Concern counsellor might feel negatively about you (14%) 3. None (13%) 4. Confidentiality/privacy/trust (9%) 5. Concern about what other people might think of you seeing a counsellor (8%) 6. Negative feelings/self/perceptions (7%)   *Mental Health Specialist/Service*   1. Too embarrassed/shy (23%) 2. None (18%) 3. Concern specialist might feel negatively about you (10%) 4. Negative feelings/self/perceptions (6%) 5. Confidentiality/privacy/trust (6%) 6. Illness/symptoms themselves (6%) 7. Cost of seeing specialist (5%) 8. Concern that what the specialist might say is wrong (5%) |  |
| **10** | **Aisbett [20]** | **2007** | AUS | 15-17 | Rural adolescents | No -Depression/anxiety | 3 | F | Community | Interview | 1. Transport/travel (accessibility) 2. Availability of qualified professionals 3. Hours of operation/waiting lists 4. Social stigma 5. Self stigma 6. Fear of social stigma 7. Exclusionary practices/ostracism 8. Social visibility 9. Gossip networks |  |
| **11** | **Biddle [21]** | **2007** | UK | 16-24 | Young adults | No -Mentally distressed | 23 | M/F | Community | Interview | 1. Questioning whether distress just “normal” or bad enough to be real 2. Avoiding acknowledging “real distress because of the stigma and permanence associated with it 3. Normalisation of symptoms 4. Shifting the threshold for “real” distress 5. Viewing the act of seeking help as making distress “real” |  |
| **12** | **Gilchrist [2429]** | **2006** | AUS | 16-24 | Young adults | No – Emotional stress (and suicide) | 21 | M/F | Community | Interview | 1. ‘Trust’ and ‘confidentiality’ 2. Stigma – ‘scared’ about what others would think of them, feel ‘uncool’ or ‘weak’ or ‘judged’ within peer/community networks 3. Friends, community members, family not having the skills to cope with the young people’s problems or not recognising (or ignoring) the vital ‘signs and indicators’ 4. Lack of knowledge of available services |  |
| **13** | **Boyd [23]** | **2007** | AUS | 17-21 | Rural psychology undergrad students | No - Mental health issue | 6 | M/F | University | Interview | 1. Lack of anonymity 2. Culture of self-reliance 3. Uninformed about the availability of services 4. Thinking GPs were not the appropriate help source for mental health problems 5. Stigma attached to the act of help-seeking |  |
| **14** | **Chew-Graham [25]** | **2003** | UK | -- | Medical students years 3-5 | Yes | 22 | M/F | University | Interview | 1. Stigma associated with ‘stress’ or ‘mental illness’ 2. Shame and embarrassment in admitting to weakness 3. Fear of confiding in a tutor 4. Fear that problems would not be treated confidentially 5. Concern that admitting to problems as an undergraduate would affect their future career as a doctor 6. Fear that confiding in clinical tutors as a student might affect future job opportunities |  |
| **15** | **Boey [22]** | **1999** | China | 18-24 | University students | Yes | 326 | M/F | University | Survey | 1. Able to solve distress on one’s own 2. Distrustful of psychiatrists 3. Problem not serious enough 4. Other alternatives available 5. Stigmatisation, face-losing, shameful 6. Consultation itself stressful 7. Setting terrifying 8. Not willing to self disclose |  |

***Note: Author****=First author;* ***Year=****Published year of study;* ***Loc****=Location of study, AUS=Australia, USA= United States of America, UK= United Kingdom;* ***Age****=Age of participants;* ***Population=****Participant group characteristics;* ***Universal****=Was the study universal (e.g., sample of all students not indicated by symptoms or diagnosis)?;* ***N****=Total number of participants;* ***Sex=****Gender of participants, , M=Male, F=Female;* ***Setting=****Where was the study recruited from?;* ***Method****=Study methodology used; NR=Not reported.*
