# Supplementary material for: Perceived barriers and facilitators to mental health help-seeking in young people: a systematic review
Source: BMC Psychiatry. 2010 Dec 30;10:113. doi: 10.1186/1471-244X-10-113 (PMC3022639; doi:10.1186/1471-244X-10-113)
Supplement: Additional file 4 — Quantitative studies included in the review. [file 1471-244X-10-113-S4.DOC]

**Quantitative studies included in the review**

**Table 4.1. Included quantitative studies on barriers (n=7)**

| **#** | **Author** | **Year** | **Loc** | **Age** | **Population** | **Universal** | **N** | **Sex** | **Setting** | **Measure used** | **Barriers listed by study** |
| --- | --- | --- | --- | --- | --- | --- | --- | --- | --- | --- | --- |
| **16** | **Sheffield [35]** | **2004** | AUS | 15-17 | High school students | Yes | 254 | M/F | High school | Participants asked what would stop them seeking help from a source (school counsellor, doctor, psychologist/psychiatrist if they were to experience a mental illness. | ***Percentage of participants endorsing barrier (%) by source type (~% estimates from graph provided):***  *School counsellor*   1. Prefer to handle myself (45%) 2. Don’t think they can help (27%) 3. Not confidential (24%) 4. Don’t want parents to know (16%) 5. Worry what others will think (14%) 6. Don’t know where to find (6%) 7. Asked but didn’t get help (5%) 8. Too far away (2%) 9. Too expensive (1%)   *Doctor*   1. Too expensive (25%) 2. Prefer to handle myself (23%) 3. Don’t think they can help (22%) 4. Don’t want parents to know (16%) 5. Not confidential (7%) 6. Worry what others will think (7%) 7. Don’t know where to find (5%) 8. Too far away (4%) 9. Asked but didn’t get help (3%)   *Psychologist/Psychiatrist*   1. Too expensive (50%) 2. Don’t know where to find (28%) 3. Prefer to handle myself (22%) 4. Don’t want parents to know (12%) 5. Too far away (10%) 6. Don’t think they can help (7%) 7. Worry what others will think (5%) 8. Not confidential (3%) 9. Asked but didn’t get help (2%) |
| **17** | **Dubow [26]** | **1990** | USA | Grade 7-12 | Semi-rural high school students | No – Students experiencing a “pressing health concern” (e.g., trouble with parents, depression) | NR -total n of 1384 | M/F | High school | For each of their three most pressing health concerns, participants were asked to indicate if they had not sought help, the reasons that prevented them from doing so from a 7 item list. | ***Percentage of participants endorsing barrier (%)***. Results for depression only are presented  *Depression*   1. I felt that no person or helping service could help (55%) 2. The problem was too personal to tell anyone (53%) 3. Concern that family members would find out (36%) |
| **18** | **West [37]** | **1991** | USA | Grade 12 | Senior high school students (grade 12) | Yes | 235 | M/F | High school | Participants were asked to read barrier items from a list and asked to rate them according to if it was a reason they did not go to see their counsellor at one or more times (barriers could be marked as Strong, Moderate, Weak, or Does not apply). | ***Percentage of participants endorsing barrier (%)***.   1. I do not like to tell a stranger about personal things (29.4%) 2. I am afraid counselor will pass information about me to other people (18.3%) 3. I did not have the time (15.7%) 4. I would be embarrassed to reveal my real concerns (15.7%) 5. Counselor was busy or not in (15.3%) 6. I’m not sure how to begin talking to the counselor (14.5%) 7. Counselor deals only with school/college/occupational problems (14%) 8. I wanted to see the counselor but never got around to it (14%) 9. It is difficult for me to talk to the counsellor (13.2%) 10. Counselor cannot help me with my real problems (12.8%) 11. Counselor has too many students to be effectively concerned with individuals (12.8%) 12. My concern was not important enough to bother the counsellor (12.3%) 13. Counselor is unable or unwilling to change schedule around my needs (11.5%) 14. I would not want my friends to know I was seeing a counsellor (10.2%) 15. Counselor prefers college-bound students (8.9%) 16. I am afraid the counselor would approve if the truth were known (8.9%) 17. Counselor has no business in my life (8.9%) 18. There is no way anyone can help the mess I’m in (8.9%) 19. I would have heard the same things I’ve heard from others (7.2%) 20. I didn’t know who my counselor was (6.8%) 21. Counselor would argue against what I want (6.8%) 22. It’s hard to talk to counselors of the opposite sex (6.8%) 23. Counselor makes me feel dumb (6.8%) 24. I had a bad experience with a counselor (6.8%) 25. Counselor seems artificial (6.8%) 26. Weak people go to counselors (6.4%) 27. Counselor might act concerned but inside will be laughing at me (6.0%) 28. I was afraid I would not get the answers I wanted to hear (6.0%) 29. I couldn’t work up the nerve (6.0%) 30. Counselor seems too superior (6.0%) 31. Counselor doesn’t understand me (6.0%) 32. I was afraid parents would find out I had talked with the counsellor (6.0%) 33. Counselor prefers student with good grades (5.5%) 34. Counselor is on the side of the teacher (5.5%) 35. I don’t trust counsellors (5.5%) 36. I felt that talking to a counselor would only increase my troubles (5.5%) 37. Counselor usually tries to rush me off (5.1%) 38. Counselor tries to run things too much (5.1%) 39. I’m too shy (4.7%) 40. I’m unsure if I can go to counselor other than one to who I am assigned (4.7%) 41. Counselor talks too much—does not listen to me (4.3%) 42. Counselor doesn’t have the information I need (4.3%) 43. If were honest, it might hurt counselor’s recommendation for my college admission or job reference (4.3%) 44. I just don’t like my counsellor (3.8%) 45. Counselor does not welcome me (3.4%) 46. It’s hard for me to talk to counsellors of the same sex (3.4%) 47. I’m afraid counselor would place me in higher level classes (2.6%) 48. I’ve seen my counselor too much already (2.1%) 49. Counselor is not interested in me (2.1%) 50. Counselor and I irritate each other (1.3%) |
| **19** | **Kuhl [32]** | **1997** | USA | Mean =15.7 | High school students | Yes | 280 | M/F | High school | Participants were asked to indicate their agreement to items on the Barriers to Adolescents Seeking Help (BASH) scale (1-6, where 1 = strongly agree, 6 = strongly disagree). | **Participants’ mean ratings of the BASH items.**  *Note: Negative items reversed scored, so that higher scores indicate higher barriers to help-seeking:*   1. If I had a problem I would solve it by myself (3.87) 2. I think I should work out my own problems (3.79) 3. Adults really can’t understand the problems that kids have (3.77) 4. If I had a problem, my family would help me more than a therapist (3.77) 5. If I had a problem, my friends would help me more than a therapist (3.67) 6. From what I know, most people get help from therapy (3.53) 7. I would never want my friends to know that I was seeing a therapist (3.45) 8. If I had a problem, my parents would think that speaking to a therapist was a good idea (3.11) 9. I cannot imagine having a problem so serious I would go for help (3.11) 10. I know people who have been helped by therapy (3.08) 11. Even if I had a problem, I’d be too embarrassed to talk to a therapist about it (2.99) 12. Even if I wanted to I would have time to see a therapist (2.96) 13. Therapists are more helpful to adults than to teenagers (2.92) 14. I know where I could find a therapist if I needed one (2.90) 15. People don’t need therapists to help them with their problems (2.90) 16. My friends would think I was crazy if I saw a therapist (2.82) 17. If I ever talked to a therapist about personal things, I’m sure my family would hear about it (2.80) 18. Therapists can’t really understand teenager’s problems today (2.79) 19. the idea of going to a therapist is pretty scary to me (2.75) 20. Going to a therapist means you don’t have the strength to handle the problem yourself (2.66) 21. If I went to a therapist it would make me feel like I was crazy (2.57) 22. I have had problems in the past which really upset me (2.55) 23. My problems will go away by themselves (2.52) 24. Therapy can often help teenagers with problems (2.40) 25. I’d never want my family to know I was seeing a therapist (2.40) 26. No matter what I do it will not change the problems I have (2.37) 27. A therapist might make me do or say something that I don’t want to (2.35) 28. I could not afford to see a therapist even if I wanted to (2.26) 29. I think that therapists really want to help people (2.23) 30. My parents have said they really don’t believe in therapy (2.15) 31. If I ever went to a therapist, my parents would be pretty upset (2.15) 32. I think therapy can be harmful (2.11) 33. If I saw a therapist my family would think I was weak (2.05) 34. If I went to see a therapist, I might find out I was crazy (1.99) 35. If I had a problem and told a therapist, he would not keep it a secret (1.98) 36. My family thinks that anyone who goes to a therapist is crazy (1.80) 37. People who see therapists are crazy (1.70)   *Listed by category of barriers*   1. Family sufficient to help (3.75) 2. Peers sufficient to help (3.67) 3. Self-sufficiency (3.26) 4. Time availability (2.93) 5. Knowledge of resources (2.91) 6. Alienation (2.86) 7. Usefulness of therapy (2.78) 8. Self-awareness/self-perception (2.52) 9. Perception of therapist (2.49) 10. Locus of control (2.41) 11. Stigma (2.40) 12. Confidentiality (2.39) 13. Affordability (2.26) |
| **20** | **Wilson [38]** | **2008** | AUS | Mean =15.4 | High school students | Yes | 118 | M/F | High School | Participants were asked to indicate their agreement to items on the Barriers to Engagement in Treatment Screen (BETS) scale (0-3, where 0 = agree, 3 = disagree). | **Barrier means for BETS items.** *Results presented are for non-treatment group only at post intervention. Higher scores indicate higher barriers to help-seeking:*   1. I feel comfortable talking to a GP who I don’t know (1.65) 2. I’m not embarrassed to talk about my problems (1.51) 3. I believe a GP can understand my thoughts and feelings (1.44) 4. I’m not worried about telling a GP how I truly feel (1.34) 5. What I think and how I feel emotionally are important enough to talk to a GP about (1.25) 6. Getting a GP’s help means I don’t have to work out my problems alone (1.15) 7. I’m happy about my family know if I’ve visited a GP (0.96) 8. I know what to expect when I go to see a GP (0.95) 9. I believe a GP has time to listen to my problems (0.88) 10. I think GPs are interested in emotional problems as well as physical health problems (0.88) 11. If I tell a GP about my personal-emotional problems, I believe they will keep it a secret (0.60) |
| **21** | **Eisenberg [27]** | **2007** | USA | 18-31+ | University students with positive PHQ (patient health questionnaire for major depression/anxiety) screens and no help-seeking | No – Depression/Anxiety | 294 | M/F | University | Participants asked to select barriers to mental health service utilisation they had experienced in the past year. | ***Percentage of students endorsing barrier (%):***   1. Stress is normal in graduate school (51%) 2. Have not had any need (45%) 3. The problem will get better by itself (37%) 4. I don’t have time (32%) 5. I don’t think anyone can understand my problems (20%) 6. I worry what others will think of me (20%) 7. I question the quality of my options (16%) 8. Other (16%) 9. I am concerned about privacy (16%) 10. I worry that my actions will be on my academic record (10%) 11. Haven’t had the chance but plan to go (10%) 12. I worry that someone will notify my parents (9%) 13. There are financial reasons (8%) 14. Had bad experiences with medication and/or therapy (7%) 15. Providers aren’t sensitive enough to cultural issues (5%) 16. I fear being hospitalized (4%) 17. The hours are inconvenient (3%) 18. Waiting time until I can get an appointment is too long (3%) 19. I have a hard time communicating in English (2%) 20. The location is inconvenient (2%) 21. No barriers that I can think of (2%) 22. Providers aren’t sensitive enough to sexual identity issues (2%) 23. The number of sessions is too limited (1%) |
| **22** | **Brimstone [24]** | **2007** | AUS | Mean =21.2 | Medical students | Yes | 71 | M/F | University | Depression vignette followed by 16 statements about concerns relating to seeking mental health care (1-4, 1 = Strongly disagree, 4 = Strongly agree). Scores of 2+ (i.e., agree or strongly agree) indicate item is a barrier. | ***Statements rating as ≥ 2 (agree or strongly agree):***   1. Worries about either knowing the doctor/counsellor or having to have future dealings with the counsellor/psychologist or general practitioner at university health care centre 2. Worries about either knowing the doctor/counsellor or having to have future dealings with the counsellor/psychologist or general practitioner at non-university health care centre |

***Note: Author****=First author;* ***Year****=Published year of study;* ***Loc****=Location of study, AUS=Australia, USA=United States of America, UK= United Kingdom;* ***Age****=Age of participants;* ***Population=****Participant group characteristics;* ***Universal****=Was the study universal (e.g., sample of all students not indicated by symptoms or diagnosis)?;* ***N****=Total number of participants;* ***Sex=****Gender of participants, M=Male, F=Female;* ***Setting=****Where was the study recruited from?. All quantitative studies used survey methodology; NR=Not reported.*
