# Supplementary material for: Perceived barriers and facilitators to mental health help-seeking in young people: a systematic review
Source: BMC Psychiatry. 2010 Dec 30;10:113. doi: 10.1186/1471-244X-10-113 (PMC3022639; doi:10.1186/1471-244X-10-113)
Supplement: Additional file 5 — Thematic analysis of qualitative studies. [file 1471-244X-10-113-S5.DOC]

**Thematic analysis of qualitative studies**

1. **Barrier themes raised by qualitative study participants (n=13)**

Thirteen studies [20-23, 25, 28, 29, 31, 34, 36, 39-41] were included in the thematic analysis of barriers in the qualitative studies (see Table 5.1). Two qualitative studies [30, 33], which focused exclusively on *characteristics* of school-based providers that may act as barriers to help-seeking were excluded from this analysis.

**Table 5.1**

| **#** | **Primary Author** | **Year** |
| --- | --- | --- |
| 1 | Wilson | 2007 [39] |
| 2 | Timlin-Scalera | 2003 [36] |
| 3 | Francis | 2006 [28] |
| 4 | Wilson | 2001 [40] |
| 5 | Lindsey | 1998 (excluded in this analysis) [33] |
| 6 | Helms | 2003 (excluded in this analysis) [30] |
| 7 | Wisdom | 2006 [41] |
| 8 | Lindsey | 2006 [34] |
| 9 | Jorm | 2007 [31] |
| 10 | Aisbett | 2007 [20] |
| 11 | Biddle | 2007 [21] |
| 12 | Boyd | 2007 [23] |
| 13 | Gilchrist | 2006 [29] |
| 14 | Chew-Graham | 2003 [25] |
| 15 | Boey | 1999 [22] |

**A total of 13 themes were identified in these studies:**

1. Public, perceived and self-stigmatising attitudes to mental illness
2. Difficulty identifying the symptoms of mental illness
3. Confidentiality and trust
4. Concern about the characteristics of the provider
5. Knowledge about mental health services
6. Fear or stress about the act of help-seeking or the source of help itself
7. Reliance on self, do not want help
8. Lack of accessibility, e.g., time, transport, cost
9. Difficulty or an unwillingness to express emotion
10. Do not want to burden someone else
11. Prefer other sources of help (e.g., family, friends)
12. Worry about effect on career
13. Others not recognising the need for help or not having the skills to cope

The studies in each theme are shown below using their study number as listed in Table 5.1 above**.** The terminology used by the study authors was preserved where possible in the descriptions below.

**1: Public, perceived and self-stigmatising attitudes to mental illness**

1. Social stigma [20]
2. Self stigma [20]
3. Fear of social stigma [20]
4. Stigma – shame, embarrassment and exclusion [33]
5. Lack of anonymity [29]
6. Stigma attached to the act of help-seeking [29]
7. Stigma associated with ‘stress’ or ‘mental illness’ [25]
8. Shame and embarrassment in admitting to weakness [25]
9. Stigma of being weak and troubled [36]
10. Stigmatisation, face-losing, shameful [22]
11. Embarrassed [39]
12. Exclusionary practices/ostracism [20]
13. Social visibility [20]
14. Gossip networks [20]
15. Pride [33]
16. Too embarrassed/shy [31]*
17. Negative feelings/self perceptions [31]*
18. Concern about what others might think [31]*
19. Concern about what the doctor/counsellor might think [31]*
20. Denial/pride [31]*
21. Exclusionary social practices [28]
22. Fear of social stigma [28]
23. Public stigma [28]
24. Self-stigma [28]
25. Lack of anonymity in rural towns [28]
26. Stigma – ‘scared’ about what others would think of them, feel ‘uncool’ or ‘weak’ or ‘judged’ within peer/community networks [23]

**2: Confidentiality and trust**

1. Fear that problems would not be treated confidentially [25]
2. Confidentiality or that others would find out [39]
3. Confidentiality or could not be trusted [39]
4. Trust [36]
5. Distrustful of psychiatrists [22]
6. Confidentiality/privacy/trust [31]
7. Confidentiality and trust [23]
8. ‘Trust’ and ‘confidentiality’ [23]

**3: Difficulty identifying the symptoms of mental illness**

1. Questioning whether distress just “normal” or bad enough to be real [21]
2. Avoiding acknowledging “real distress because of the stigma and permanence associated with it [21]
3. Normalisation of symptoms [21]
4. Shifting the threshold for “real” distress [21]
5. Problem not serious enough [22]
6. Problems did not warrant professional intervention [39]
7. Lack of insight into their own problems and need for services [36]
8. Desire to be normal, e.g., reject diagnosis, normalise symptoms [41]

**4: Lack of accessibility, e.g., time, transport, cost**

1. Hours of operation/waiting lists [20]
2. Availability of qualified professionals [20]
3. Transport/travel (accessibility) [20]
4. Too busy/No time to seek help [39]
5. Cost of seeing specialist [31]*
6. Lack of specialist local services [28]
7. Lack of transport to services located out of town [28]

**5: Concern about characteristics of provider**

1. Race of the provider being different to their own [33]
2. Perceptions of mental health staff as being inaccessible, unavailable and unfamiliar [36]
3. Did not want to talk to someone who was a stranger [41]
4. Could not effectively help [39]
5. Concern that what the doctor/counsellor says is wrong [31]
6. A lack of connection with the provider, e.g., they were not competent, do not listen [41]

**6: Reliance on self, do not want help**

1. Handle problem on own [33]
2. Culture of self-reliance [29]
3. Culture of self-reliance [28]
4. Able to solve distress on one’s own [22]
5. Rely on oneself rather than seek help [39]
6. Would not want to [39]

**7: Knowledge about mental health services**

1. Uninformed about the availability of services [29]
2. Lack of awareness and understanding of services [36]
3. A lack of education [40]
4. Thinking GPs were not the appropriate help source for mental health problems [29]
5. Lack of knowledge of available services [23]

**8: Fear or stress about the act of help-seeking or source of help itself**

1. Viewing the act of seeking help as making distress “real” [21]
2. Fear of confiding in a tutor [25]
3. Consultation itself stressful [22]
4. Setting terrifying [22]
5. Afraid [39]

**9: Difficulty or an unwillingness to express emotion**

1. Expression of emotions viewed as sign of weakness [33]
2. Not willing to self disclose [22]
3. Illness/symptoms themselves [31]

**10: Do not want to burden someone else**

1. Did not want to burden someone else with their problems [36]
2. Did not want others to take care of them [36]
3. Desire to be autonomous, e.g., not having a voice in treatment, getting little information about what was happening [41]

**11: Prefer other sources of help (e.g., family, friends)**

1. Other alternatives available [22]
2. Prefer a friend or family member [39]

**12: Worry about effect on career**

- 1. Concern that admitting to problems as an undergraduate would affect their future career as a doctor [25]
  2. Fear that confiding in clinical tutors as a student might affect future job opportunities [25]

**13: Others not recognising the need for help or not having the skills to cope**

- - 1. Friends, community members, family not having the skills to cope with the young people’s problems or not recognising (or ignoring) the vital ‘signs and indicators’ [23]

Note: *Some items for Jorm (2007) [31] were consolidated into the barrier themes for all sources of help (i.e., General practitioner, counsellor, mental health specialist/service).

- - 1. **Facilitator themes raised by qualitative study participants (n=3)**

Three studies [34, 36, 40] were included in the thematic analysis of barriers in the qualitative studies (see Table 5.2 below). The study by Lindsey (1998) [33] was excluded as it focused exclusively on *characteristics* of school-based adults that may act as facilitators to help-seeking.

**Table 5.2**

| **#** | **Author** | **Year** |
| --- | --- | --- |
| 1 | Timlin-Scalera | 2003 [36] |
| 2 | Wilson | 2001 [40] |
| 3 | Lindsey | 1998 (excluded in this analysis) [33] |
| 4 | Lindsey | 2006 [34] |

**A total of 7 themes were identified in these studies:**

1. Positive past experiences with help-seeking
2. Social support or encouragement from others
3. Confidentiality and trust in the provider
4. Education and awareness
5. Perceiving the problem as serious
6. Ease of expressing emotion and openness
7. Positive attitudes towards seeking help

The studies in each theme are shown below using their study number as listed in Table 5.2 above**.** The terminology used by the study authors was preserved where possible in the descriptions below.

**1: Positive past experiences with help-seeking**

1. Having previous positive encounters with the mental health field [36]
2. Knowledge from prior help [40]
3. Successful prior help-seeking [40]
4. A strong and open relationship with a potential help-giver [40]
5. How well the provider treated and engaged them [34]

**2: Social support or encouragement from others**

1. Someone suggesting it [36]
2. Problem normalisation (validation/normalising of problem by others) [40]

**3: Confidentiality and trust**

1. A perception of assured confidentiality [36]
2. Primarily a matter of trust [40]

**4: Education and awareness**

1. Knowledge from peer discussion [40]

**5: Perceiving the problem as serious**

1. Having what they perceived to be as a very serious problem [36]

**6: Ease of expressing emotion and openness**

1. A culture of openness in their homes [36]

**7: Positive attitudes towards seeking help**

1. Positive attitudes towards seeking help [40]
